# On the Use of Ti_3_C_2_*T*_*x*_ MXene as a Negative Electrode Material for Lithium-Ion Batteries

**DOI:** 10.1021/acsomega.2c05785

**Published:** 2022-11-07

**Authors:** Tatiana Koriukina, Antonia Kotronia, Joseph Halim, Maria Hahlin, Johanna Rosen, Kristina Edström, Leif Nyholm

**Affiliations:** † The Ångström Advanced Battery Center, Department of Chemistry-Ångström Laboratory, Uppsala University, P.O. Box 538, SE-751 21 Uppsala, Sweden; ‡ Materials Design Division, Department of Physics, Chemistry and Biology (IFM), Linköping University, 58183 Linköping, Sweden

## Abstract

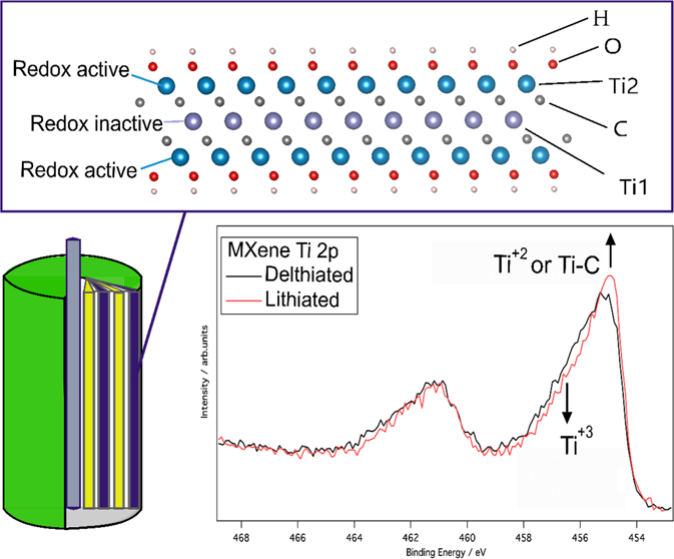

The pursuit of new and better battery materials has given rise to numerous studies of the possibilities to use two-dimensional negative electrode materials, such as MXenes, in lithium-ion batteries. Nevertheless, both the origin of the capacity and the reasons for significant variations in the capacity seen for different MXene electrodes still remain unclear, even for the most studied MXene: Ti_3_C_2_*T*_*x*_. Herein, freestanding Ti_3_C_2_*T*_*x*_ MXene films, composed only of Ti_3_C_2_*T*_*x*_ MXene flakes, are studied as additive-free negative lithium-ion battery electrodes, employing lithium metal half-cells and a combination of chronopotentiometry, cyclic voltammetry, X-ray photoelectron spectroscopy, hard X-ray photoelectron spectroscopy, and X-ray absorption spectroscopy experiments. The aim of this study is to identify the redox reactions responsible for the observed reversible and irreversible capacities of Ti_3_C_2_*T*_*x*_-based lithium-ion batteries as well as the reasons for the significant capacity variation seen in the literature. The results demonstrate that the reversible capacity mainly stems from redox reactions involving the *T*_*x*_–Ti–C titanium species situated on the surfaces of the MXene flakes, whereas the Ti–C titanium present in the core of the flakes remains electro-inactive. While a relatively low reversible capacity is obtained for electrodes composed of pristine Ti_3_C_2_*T*_*x*_ MXene flakes, significantly higher capacities are seen after having exposed the flakes to water and air prior to the manufacturing of the electrodes. This is ascribed to a change in the titanium oxidation state at the surfaces of the MXene flakes, resulting in increased concentrations of Ti(II), Ti(III), and Ti(IV) in the *T*_*x*_–Ti–C surface species. The significant irreversible capacity seen in the first cycles is mainly attributed to the presence of residual water in the Ti_3_C_2_*T*_*x*_ electrodes. As the capacities of Ti_3_C_2_*T*_*x*_ MXene negative electrodes depend on the concentration of Ti(II), Ti(III), and Ti(IV) in the *T*_*x*_–Ti–C surface species and the water content, different capacities can be expected when using different manufacturing, pretreatment, and drying procedures.

## Introduction

MXenes constitute a novel class of two-dimensional materials which has obtained its name from the fact that they are produced from MAX phases (where M is a transition metal, A is an A-group element, and X is N and/or C)^1^ by etching away the A metal (e.g., Al).^2−8^ As a result, MXenes are 2D transition-metal nitrides or carbides with the general chemical formula M_*n*+1_X_*n*_*T*_*x*_, where *n* is 1–4, and T is the surface termination (e.g., O, OH, F, or Cl). Since their discovery in 2011,^2^ MXenes have been shown to exhibit several interesting properties, including high electronic conductivity and high surface area. MXenes are therefore considered promising with respect to applications involving energy storage, mainly as electrode materials for supercapacitors but also as negative electrode materials for lithium-ion batteries.^2,9−15^

To be used as a lithium-ion battery material, it is, however, not enough that the material has a high electronic conductivity and a high surface area. A good negative electrode material also needs to undergo a reduction during the lithiation step and an oxidation during the subsequent delithiation step. The redox reactions, which should be reversible and occur in a suitable potential region (e.g., between 2.5 and 0 V vs Li^+^/Li), should involve the full volume of the material rather than merely the surface of the material (as for a supercapacitor material). To be able to use an electrode material properly, the composition of the material, its capacity, as well as the redox reactions responsible for the capacity all need to be known. Ideally, the specific capacity of a negative electrode material should be higher than 372 mA h g^–1^, that is, the specific capacity of graphite, which is the most commonly used negative electrode material at present. Many MXene-based materials do not fulfill these requirements, at least not yet, as the origins of the obtained capacities remain unclear and as the capacities have been found to depend significantly on the experimental conditions used to manufacture the MXene materials.

Ti_3_C_2_*T*_*x*_ is an MXene that has been frequently studied as a negative lithium-ion battery material.^9,11,12,15−17^ Although the experimentally found capacity of Ti_3_C_2_*T*_*x*_ has been suggested to be due to a titanium-based lithium intercalation reaction,^9,12,15^ it is still not clear which titanium species are responsible for the obtained capacity. Moreover, as significantly different capacities have been reported for different Ti_3_C_2_*T*_*x*_-based electrodes, it can also be suspected that the capacities depend on the processes used to manufacture the MXene material and/or the employed electrodes. Freestanding Ti_3_C_2_*T*_*x*_ electrodes have, for example, been found to exhibit specific capacities of 410 mA h g^–1^ at a 1C cycling rate and 110 mA h g^–1^ at a rate of 36C,^12^ when cycling between 2.5 and 0.05 V versus Li^+^/Li. In another study, a capacity of about 35 mA h g^–1^ was, on the other hand, obtained when cycling freestanding 5 μm thick multilayered Ti_3_C_2_*T*_*x*_ electrodes at a rate of 0.5C between 3.0 and 0.01 V versus Li^+^/Li.^15^ The different capacities have been proposed to be due to different degrees of restacking of the Ti_3_C_2_*T*_*x*_ flakes inside multilayered freestanding electrodes, making it difficult to access all of the material. To circumvent this problem, approaches aimed at chemical modifications of the Ti_3_C_2_*T*_*x*_ material or structural modifications of the Ti_3_C_2_*T*_*x*_ electrodes have been developed. These have involved, for example, chemical etching,^15^ flash oxidation,^16^ and oxidation of Ti_3_C_2_*T*_*x*_ with KOH,^17^ as well as modifications of the structure of freestanding electrodes via the incorporation of carbon nanotubes.^15^ A capacity of 110 mA h g^–1^ at a rate of 0.5C was, for example, obtained^15^ after increasing the porosity of the Ti_3_C_2_*T*_*x*_ material via chemical etching. A freestanding Ti_3_C_2_*T*_*x*_ electrode containing 10 wt % of CNT^15^ was found to have a capacity of 220 mA h g^–1^ at 0.5C, whereas a capacity of 500 mA h g^–1^ at 0.5C was found when using a composite film prepared by combining the chemical etching of the Ti_3_C_2_*T*_*x*_ powder with the addition of 10 wt % of carbon nanotubes.^15^ Reversible capacities of 220 mA h g^–1^ (at a rate of C/18) have also been found after using flash oxidation in air to generate TiO_2_ particles on the surface of composite Ti_3_C_2_ powder electrodes. In the latter case, the capacity was attributed to the lithiation and delithiation of the obtained TiO_2_ anatase.^16^ These varying results, obtained with different Ti_3_C_2_*T*_*x*_ electrodes, suggest that the capacity may depend not only on the structure of the electrode but also on the oxidation state of the MXene material.

The results presented in the literature clearly show that the capacities of electrodes composed of Ti_3_C_2_*T*_*x*_ can differ significantly depending on how the electrodes were made and/or pretreated. In this context, it should be mentioned that Ti_3_C_2_*T*_*x*_ colloidal solutions are known to undergo spontaneous oxidation to finally yield TiO_2_ and carbon upon exposure to air.^18^ This indicates that oxidized titanium species, including TiO_2_, may be present on the surface of a synthesized Ti_3_C_2_*T*_*x*_ powder even if care is taken to minimize its exposure to air. This is interesting as the results discussed above suggest that the Ti_3_C_2_*T*_*x*_ capacity can be increased by oxidizing the Ti_3_C_2_*T*_*x*_ material. As was recently demonstrated,^19^ the approach used to prepare the MXene from the MAX phase can also affect the electrochemical performance of Ti_3_C_2_*T*_*x*_. Etching the MAX phase with a Lewis acid molten salt, followed by an oxidative treatment with ammonium persulfate solution resulted in a capacity of 205 mA h g^–1^ for a composite electrode containing 80 wt % MXene powder and 15 wt % carbon black.^19^ This capacity, which was obtained when cycling between 3 and 0.2 V versus Li^+^/Li at a scan rate of 0.5 mV s^–1^, was ascribed to redox reactions involving titanium.

In addition to the experimental work discussed above, studies have also focused on the calculation of the theoretical capacity and reduction potential of Ti_3_C_2_*T*_*x*_ when used as a negative electrode material in lithium-ion batteries. For Ti_3_C_2_, the results of density functional theory calculations indicated that a theoretical capacity of 320 mA h g^–1^ should be obtained based on the redox reaction Ti_3_C_2_ + 2e^–^ + 2 Li^+^ = Ti_3_C_2_Li_2_, which was calculated to have a standard potential of about 0.62 V versus Li^+^/Li.^13^ The same authors also found that surface functionalization of Ti_3_C_2_ yielding Ti_3_C_2_F_2_ or Ti_3_C_2_(OH)_2_ should result in lower theoretical capacities, that is, 130 and 67 mA h g^–1^, respectively, as well as lower standard potentials, that is, 0.56 and 0.14 V versus Li^+^/Li, respectively. Due to the lower capacities, it was concluded that surface modifications of Ti_3_C_2_ should be avoided as much as possible when using Ti_3_C_2_ as a negative electrode material in lithium-ion batteries. This recommendation is, however, not in agreement with the experimental results discussed above, indicating that the oxidation of Ti_3_C_2_*T*_*x*_ electrodes can give significantly increased capacities. Moreover, the experimental data also show that the main part of the capacity stemmed from redox reactions taking place at potentials significantly higher than 0.62 V versus Li^+^/Li. These findings indicate that the capacities obtained during the cycling of Ti_3_C_2_*T*_*x*_ electrodes are unlikely to stem from the reduction of Ti_3_C_2_*T*_*x*_ yielding Ti_3_C_2_*T*_*x*_Li_2_, as assumed in the abovementioned theoretical study. This raises questions regarding the origin of the capacity seen when cycling Ti_3_C_2_*T*_*x*_ MXene electrodes in lithium-ion batteries. In addition to this and the abovementioned issues concerning the significantly different capacities reported for different Ti_3_C_2_*T*_*x*_ electrodes, the origin of the large irreversible capacity, often seen on the first cycles, is still to be properly explained. Although the irreversible capacity typically is ascribed to the formation of a solid electrolyte interphase (SEI) layer on the electrode (mainly at potentials below 1 V vs Li^+^/Li), the experimental results clearly show that the irreversible capacity also stems from, at least one, unidentified reduction taking place at higher potentials.

The main aim of the present work is to identify the redox reactions responsible for the reversible and irreversible capacities obtained for freestanding Ti_3_C_2_*T*_*x*_ MXene electrodes, by employing a combination of chronopotentiometry, cyclic voltammetry, X-ray photoelectron spectroscopy (XPS), hard X-ray photoelectron spectroscopy (HAXPES), and X-ray absorption spectroscopy (XAS) data. The effect of spontaneous oxidation of Ti_3_C_2_*T*_*x*_ in air on the capacities of the electrodes is evaluated and compared with the changes seen in the XPS, HAXPES, and XAS data. Experiments are also conducted with Ti_3_C_2_*T*_*x*_ electrodes, prior to and after different drying steps, to evaluate the influence of the water content in the electrodes on their cycling performances. It is shown that the reversible capacities of the Ti_3_C_2_*T*_*x*_ electrodes mainly stem from redox reactions involving the *T*_*x*_–Ti–C titanium species situated on the surfaces of the MXene flakes, whereas the Ti–C titanium present within the flakes remains electrochemically inactive.

## Experimental Section

### Synthesis

The Ti_3_C_2_*T*_*x*_ MXene, which was received in the form of a suspension, was synthesized as previously described.^20^ To manufacture Ti_3_C_2_*T*_*x*_, its precursor Ti_3_AlC_2_ was first produced from a 1:1:2 molar ratio mixture of TiC (Alfa Aesar, 98+%), Ti (Alfa Aesar, 98+%), and Al (Alfa Aesar, 98+%), obtained by mixing for 5 min using a mortar and pestle. The mixture was then inserted in an alumina tube furnace with argon gas flowing through it. The furnace was heated to 1450 °C and held there for 280 min before being cooled down to room temperature. The heating and cooling rate was 5 °C min^–1^. The resulting material was a lightly sintered Ti_3_AlC_2_ sample which was then crushed into a powder, with the particle size less than 60 μm, using a mortar and pestle.

To convert Ti_3_AlC_2_ to Ti_3_C_2_*T*_*x*_ flakes, 0.5 g of Ti_3_AlC_2_ powder was added to a premixed 10 mL aqueous solution of 12 M HCl (Fisher, technical grade) and 2.3 M LiF (Alfa Aesar, 98+%) in a Teflon bottle. Prior to adding the Ti_3_AlC_2_ powder to the HCl–LiF solution, this solution was placed in an ice bath. After adding the Ti_3_AlC_2_ powder, the whole mixture was kept in the ice bath for 30 min. This was done to avoid the initial overheating that can result from the exothermic nature of the aluminum etching reaction. The Teflon bottle was then placed on a magnetic stirrer hot plate in an oil bath and held at 35 °C for 24 h. After the completion of the reaction, the mixture was washed three times with 40 mL of 1 M HCl to remove excess LiF, followed by three washings with 40 mL of 1 M LiCl (Alfa Aesar, 98+%). The mixture was subsequently repeatedly washed with 40 mL of distilled water until a dark black supernatant was observed. The resulting suspension was then centrifuged for 20 min at 2000 rpm to produce the Ti_3_C_2_*T*_*x*_ colloidal aqueous solution. Further details regarding the synthesis can be found in the article published by Ghidiu et al.^5^

Once received, the suspension was vacuum-filtrated through a 3501 coated PP Celgard membrane to obtain a Ti_3_C_2_*T*_*x*_ MXene freestanding film with a thickness of 5–7 μm. Freestanding electrodes with a diameter of either 7 mm (i.e., an area of 0.38 cm^2^) and a mass loading of about 1.3 mg cm^–2^ (i.e., 0.49 mg), or a diameter of 10 mm (i.e., an area of 0.79 cm^2^) with a mass loading of about 1.7 mg/cm^2^ (i.e., 1.31 mg) were made by punching the Ti_3_C_2_*T*_*x*_ films (see Figure S1 for a SEM cross-section image). The electrode mass loading was 0.49 mg unless stated otherwise. The electrodes were prepared as soon as the freestanding Ti_3_C_2_T_*x*_ MXene film had been manufactured, and the electrodes were then transferred into the glovebox for storage and subsequent drying. This procedure was used to minimize the exposure of the MXene to air and water in order to slow down the oxidation of the Ti_3_C_2_*T*_*x*_ MXene. Prior to the cell assembly, the electrodes were dried at 120 °C in a vacuum oven located in a glovebox for 16 h, if not stated otherwise.

### Impact of Air Exposure

In the Ti_3_C_2_*T*_*x*_ oxidation experiment, 15 mL of a Ti_3_C_2_*T*_*x*_ suspension with a concentration of 3–4 mg/mL in deionized water was left in an open vial (at room temperature), that is, exposed to air, for up to 28 days. On the 7th, 14^th^, and 28th days of the experiment, 5 mL of the suspension was taken from the vial with a syringe and filtrated to manufacture a freestanding Ti_3_C_2_*T*_*x*_ film. Electrodes were then prepared from these films as described above to yield electrodes with a mass loading of 1.5 mg cm^–2^.

### Cell Assembly and Testing

Two-electrode pouch cells containing Ti_3_C_2_*T*_*x*_ (see above) and Li (Cyprus Foote Mineral, 125 μm thick foil, punched to 11 mm diameter disks) electrodes were assembled, each containing a Celgard 2325 separator impregnated with 100 μL of 1 M LiPF_6_ in 1:1 (v/v) EC/DEC (LP40) electrolyte (Gotion, H_2_O < 14 ppm). The cells were assembled and sealed in an argon-filled glovebox (H_2_O, O_2_ < 1 ppm) using two copper strips as the current collectors.

The coin cells (CR2032) containing electrodes composed of Ti_3_C_2_*T*_*x*_ that had been in contact with air for different times in an open vial (as described above) also contained Li–metal disks with a diameter of 13 mm as combined reference and counter electrodes, as well as Celgard 2400 separators and LP40 electrolyte. These cells were assembled and sealed in an argon-filled glovebox (H_2_O, O_2_ < 1 ppm).

The cyclic voltammetry (CV) experiments, which were performed with a Biologic MPG2 instrument, were conducted by scanning the potential of the freestanding Ti_3_C_2_*T*_*x*_ electrode from 3.0 to 0 V versus Li^+^/Li and then back to 3.0 V at a scan rate of 0.1 mV s^–1^, if not stated otherwise.

Galvanostatic cycling (i.e., constant current cycling, CC) was performed with an Arbin battery tester using a current density of 10 mA g^–1^ and cutoff voltages of 0 and 3.0 V versus Li^+^/Li, respectively, unless stated otherwise.

The ac impedance experiments were performed with a Ti_3_C_2_T_*x*_/Li cell using a Biologic MPG2 instrument. The ac impedance was first measured at the OCV (∼3 V vs Li^+^/Li) and then after scanning the potential (at a scan rate of 0.1 mV s^–1^) to 1.9 V versus Li^+^/Li and 0.3 V versus Li^+^/Li and subsequently to 2.3 V versus Li^+^/Li and 3.0 V versus Li^+^/Li. The employed frequency range was 5 mHz to 20 kHz (seven points per decade were recorded), and the amplitude of the ac signal was 10 mV. The cell was held at each of the abovementioned potentials for 30 min prior to the ac measurement.

### Spectroscopy Measurements

The in-house XPS measurements were performed with a PHI 5500 X-ray photoelectron spectrometer using an Al source with Kα radiation (1486.6 eV) and an electron emission angle of 45°, a pass energy of 23.5 eV, a step size of 0.1 eV, and time per step of 100 mS. The energy calibration was performed by referencing all spectra to the C 1s peak originating from the Ti–C peak located at 282.0 eV. To study the species found on the surface of the Ti_3_C_2_*T*_*x*_ MXene freestanding electrodes after lithiation (reduction) and delithiation (oxidation), respectively, the electrodes were subjected to a CV experiment (described above), followed by ex situ XPS and HAXPES analyses. A pristine electrode, dried at 120 °C for 16 h in vacuum, and an electrode left in contact with the electrolyte under open-circuit conditions were also studied for comparison. The cycled cells were stopped either at 0.3 V vs Li^+^/Li on the lithiation (i.e., reduction) step, or at 2.3 V versus Li^+^/Li on the delithiation (i.e., oxidation) step, of the first cycle. After finishing the cycling, the cells were transferred to an argon-filled glovebox (H_2_O, O_2_ < 1 ppm), in which the electrodes were extracted and washed with dimethyl carbonate (DMC, ≥99%, Sigma-Aldrich). The electrodes were then transferred from the glovebox into the XPS machine (or HAXPES end-station) without exposure to air using an argon-filled load-lock.

The HAXPES experiments (with an X-ray beam energy of 2.35 keV) and surface-sensitive XAS measurements with a total electron yield (TEY) detector were performed at the I09 beamline, Diamond Light Source Ltd, UK. The bulk-sensitive XAS data was collected in the transmission mode at the BALDER beamline, MAX IV Laboratory, Lund, Sweden. In the XAS measurements, electrodes with a thickness of between 8 and 10 μm and a mass loading of approximately 3.5 mg cm^–2^ were employed. The latter electrodes had been dried at 300 °C under vacuum for 16 h prior to cell assembly. A titanium foil and a pellet of TiO_2_ anatase powder (Sigma-Aldrich, powder 99.8% trace metal basis) were used as references. Prior to the XAS measurements, the Ti_3_C_2_*T*_*x*_ electrodes were hermetically sealed in Kapton tape and pouch material in a glovebox to enable their inert transfer to the beamline.

## Results and Discussion

**Figure 1:**
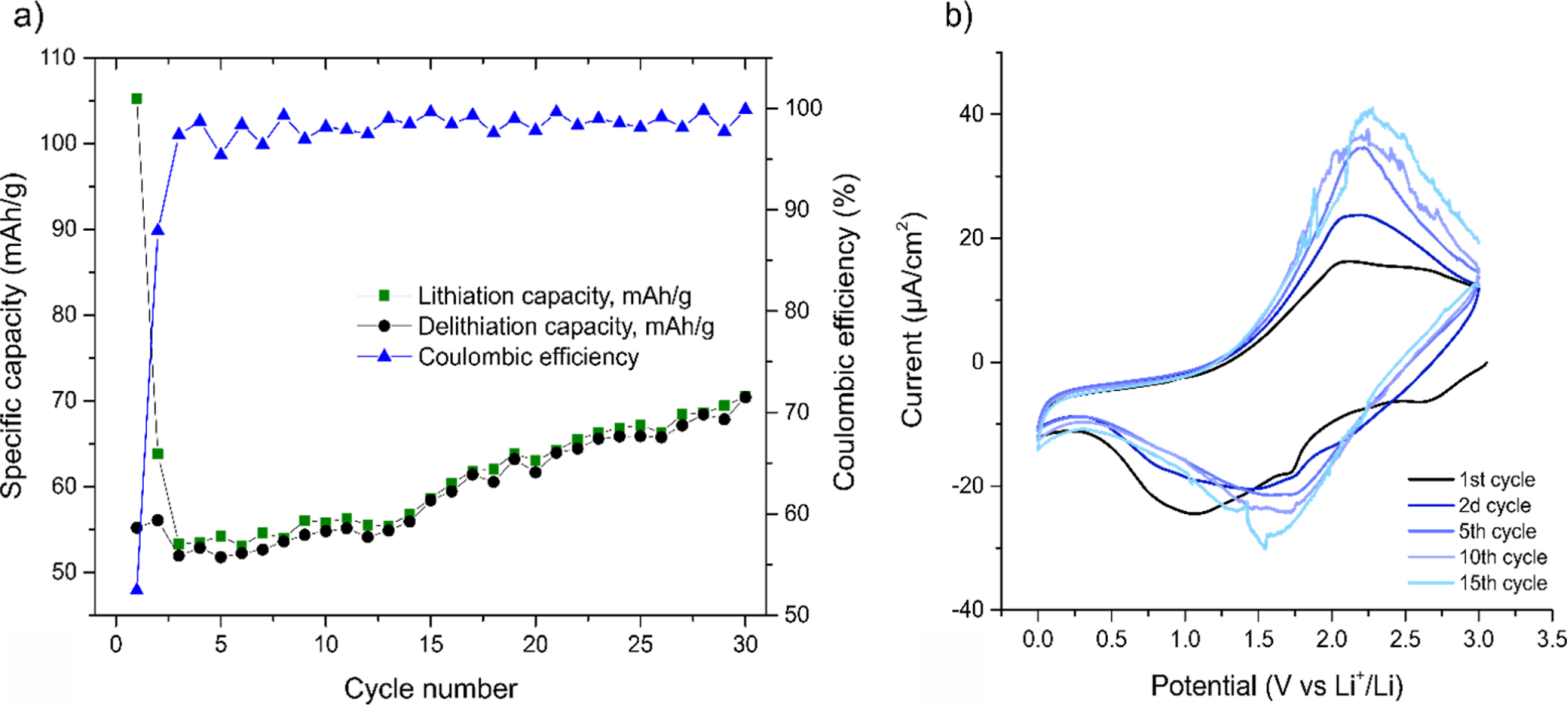
(a) Lithiation (i.e., reduction) and delithiation (i.e., oxidation) capacities as well as Coulombic efficiency as a function of the cycle number for a freestanding Ti_3_C_2_*T*_*x*_ electrode for constant current cycling with a current density of 10 mA g^–1^. (b) Cyclic voltammograms recorded for an analogous electrode at a scan rate of 0.1 mV s^–1^. The electrode mass loading was 1.31 mg.

To focus on the electrochemical performance of the Ti_3_C_2_*T*_*x*_ MXene (i.e., Ti_3_C_2_ featuring different surface groups, *T*_*x*_), the present study was conducted with freestanding, binder-free, and conductive additive-free electrodes. As the electrodes obtained via the filtering of Ti_3_C_2_*T*_*x*_ MXene suspensions only contained Ti_3_C_2_*T*_*x*_ flakes, the electrochemical behavior of the electrodes should therefore be determined by the electrochemical properties of the Ti_3_C_2_*T*_*x*_ flakes. The obtained capacities should, however, also be affected by the degree of restacking of the Ti_3_C_2_*T*_*x*_ flakes via a change of the electrochemically active surface area of the electrodes. The electrodes were first cycled between 3.0 and 0 V versus Li^+^/Li in half-cells containing lithium–metal electrodes, employing either a constant current of 10 mA g^–1^ or cyclic voltammetry at a scan rate of 0.1 mV s^–1^. As seen in Figure 1a, the 5–7 μm thick freestanding electrode was found to exhibit a lithiation capacity of about 105 mA h g^–1^ on the first constant current cycle, whereas the corresponding value was about 64 mA h g^–1^ on the second cycle (see Table S1 in the Supporting Information). From the third cycle onward, the lithiation capacity generally increased to reach a value of about 68 mA h g^–1^ after 27 cycles. The cycling curves are shown in Figure S2 in the Supporting Information. The initial capacity loss and the first-cycle Coulombic efficiency of less than 50% clearly indicate the presence of significant irreversible capacity. The first-cycle voltammetric lithiation capacity (see Figures 1b and S3) was also about 2.4 times larger than the corresponding delithiation capacity. In the voltammetric cycling, the first- and second-cycle lithiation capacities were about 75 and 65 mA h g^–1^, respectively. In analogy with the constant current results, the voltammetric lithiation and delithiation capacities increased after the third cycle (see Figure S3), yielding a lithiation capacity of about 73 mA h g^–1^ after 15 cycles.

The experimental results hence show that the specific capacities for the Ti_3_C_2_*T*_*x*_ electrodes were significantly lower than that of graphite (i.e., 372 mA h g^–1^), which typically is used as the negative electrode material in lithium-ion batteries. As is explained in the Supporting Information, the capacities were, however, too high to be compatible with the Ti_3_C_2_*T*_*x*_ MXene double-layer capacity, assuming a nitrogen BET surface area of about 20 m^2^ g^–1^.^15^ This indicates that the main part of the capacity stemmed from, at least, one redox couple. This hypothesis is further supported by the shapes of the voltammograms in Figure 1b which indicate that the capacity stemmed from a broad lithiation (i.e., reduction) peak at about 1.6 V versus Li^+^/Li and a broad delithiation (i.e., oxidation) peak at about 2.2 V versus Li^+^/Li. One possibility could then be a reduction corresponding to those previously suggested for Ti_3_C_2_, Ti_3_C_2_F_2_, and Ti_3_C_2_OH_2_ (yielding Ti_3_C_2_Li_2_, Ti_3_C_2_F_2_Li_2_, and Ti_3_C_2_OH_2_Li_2_, respectively). The standard potential and capacity for the reaction Ti_3_C_2_ + 2e^–^ + 2 Li^+^ = Ti_3_C_2_Li_2_ have been estimated to be 0.62 V versus Li^+^/Li and 320 mA h g^–1^, respectively,^13^ whereas the corresponding values for the analogous reductions of Ti_3_C_2_F_2_ and Ti_3_C_2_OH_2_ were reported to be 0.56 V versus Li^+^/Li and 130 mA h g^–1^ and 0.14 V vs Li^+^/Li and 67 mA h g^–1^, respectively. These reactions are, however, not compatible with the second and subsequent cycle voltammograms (see Figure 1b), all featuring a lithiation peak at about 1.6 V versus Li^+^/Li and a delithiation peak at about 2.2 V versus Li^+^/Li. The experimental results consequently give rise to several questions: What were the reversible capacities due to? Why were the reversible capacities so relatively low? What was causing the initial irreversible capacity loss?

To answer the first two questions, one should first consider the redox reactions that may take place in the studied potential region. These redox reactions should either involve the carbon or titanium in the Ti_3_C_2_*T*_*x*_ MXene flakes. The hypothesis that the lithiation peaks seen at about 1.6 V versus Li^+^/Li were due to the reduction of the carbon in the Ti_3_C_2_*T*_*x*_ MXene can, however, be rejected as the carbon should be reduced at significantly lower potentials. The standard potential for the (carbon reduction) reaction 2*C* + 2e^–^ + 2Li^+^ = Li_2_C_2_ should be about 0.3 V versus Li^+^/Li (see the Supporting Information). This, incidentally, also indicates that it is the carbon that is reduced in the abovementioned reductions of Ti_3_C_2_, Ti_3_C_2_F_2_, and Ti_3_C_2_OH_2_. Due to the low carbon reduction potential, carbon can clearly not oxidize titanium (as carbon is a very weak oxidizing agent). Both the carbon and titanium present in Ti_3_C_2_ must, therefore, be elemental (see the discussion in the Supporting Information), which is why Ti_3_C_2_ should be electrochemically inactive, at least at potentials above about 0.62 V versus Li^+^/Li.

**Figure 2:**
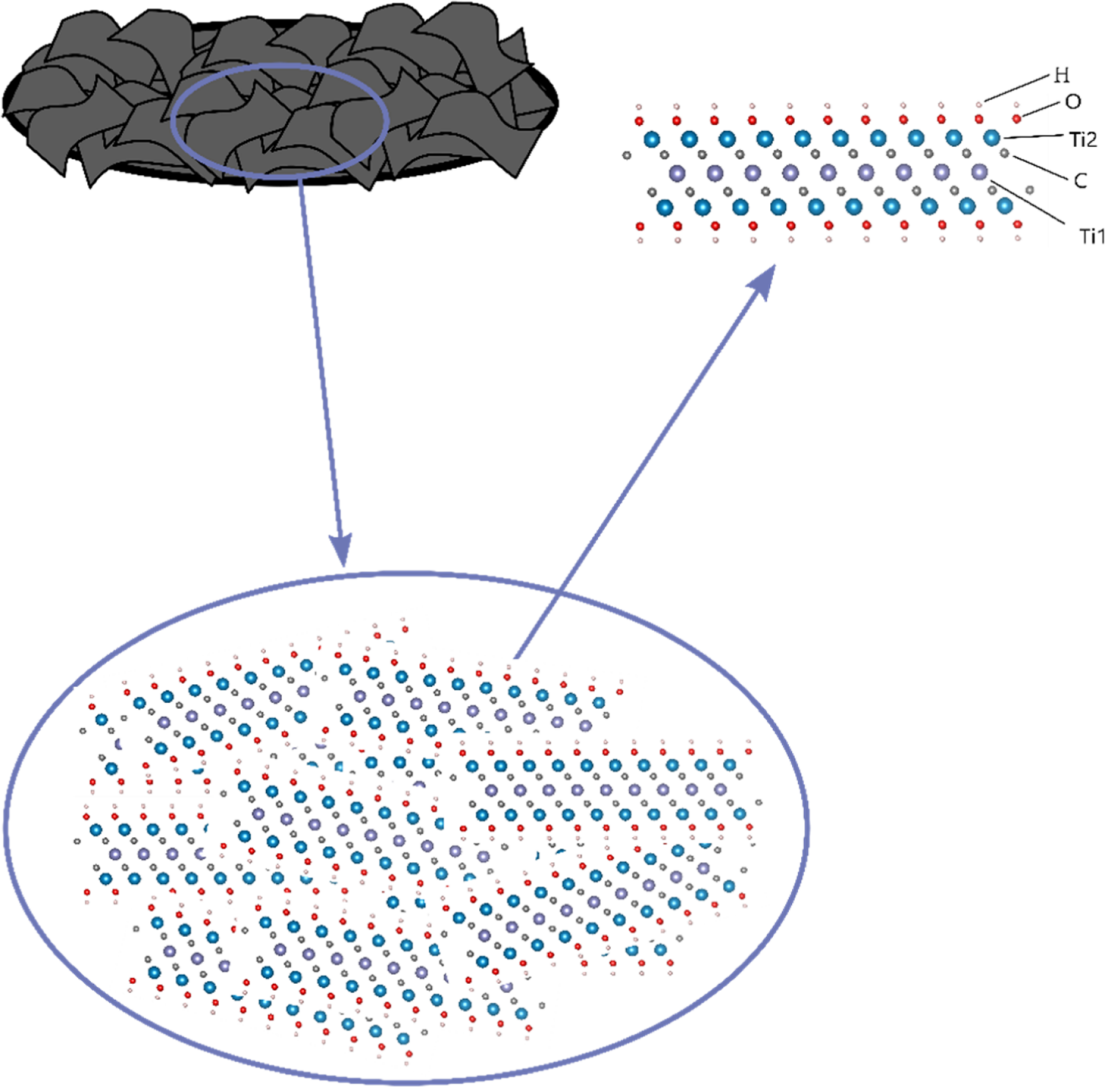
Schematic illustrations depicting a freestanding Ti_3_C_2_*T*_*x*_ electrode (top left), a magnification of the Ti_3_C_2_*T*_*x*_ flakes within the bulk of the electrode (bottom) as well as the structure of an individual Ti_3_C_2_*T*_*x*_ MXene flake (top right). Ti1 denotes the titanium in the Ti–C layer, whereas Ti2 denotes the titanium in a *T*_*x*_–Ti–C layer.

At this point, it should, however, be recalled that the employed freestanding electrodes contained Ti_3_C_2_*T*_*x*_ (rather than Ti_3_C_2_) flakes as the Ti_3_C_2_ flakes produced in the Ti_3_C_2_*T*_*x*_ manufacturing process should undergo a spontaneous reaction with water and/or oxygen.^2^ As this reaction in fact involves an oxidation of the titanium at the surface of each Ti_3_C_2_ flake, the obtained Ti_3_C_2_*T*_*x*_ flake will then contain two types of titanium species, the *T*_*x*_–Ti–C titanium species situated on the surfaces of the flakes and the Ti–C present at the center of the flakes, as is schematically illustrated in Figure 2. While the titanium in *T*_*x*_–Ti–C bonds both to carbon and the *T*_*x*_ surface group, the titanium in Ti–C thus only bonds to carbon. In the discussion below, these two titanium species will therefore be referred to as *T*_*x*_–Ti–C and Ti–C, respectively. Whereas the oxidation state of the titanium in *T*_*x*_–Ti–C should be higher than zero, Ti–C titanium should remain elemental. The fact that Ti_3_C_2_ flakes should undergo spontaneous oxidation upon exposure to air and/or water should not come as a surprise as it is well-known that titanium carbides undergo an oxidation, finally yielding titanium dioxide and carbon^15,18,21^ when exposed to oxygen and/or water (see the Supporting Information). As it is very difficult to completely avoid such exposure of the Ti_3_C_2_ flakes, it is reasonable to assume that the observed lithiation capacity could stem from reductions involving *T*_*x*_–Ti–C titanium species. This hypothesis is further supported by the fact that the activation of MXenes using surface oxidation has been found to enhance their reversible capacities.^16^ With respect to the relative low capacities seen in Figure 1, it is, however, also important to recall that the Ti_3_C_2_*T*_*x*_ MXene flakes used in the present electrodes had been protected as much as possible from contact with both air and water during the synthesis, electrode manufacturing, and battery assembly. It could therefore be expected that the average oxidation state of the *T*_*x*_–Ti–C titanium species was relatively low for the electrodes investigated here.

**Figure 3:**
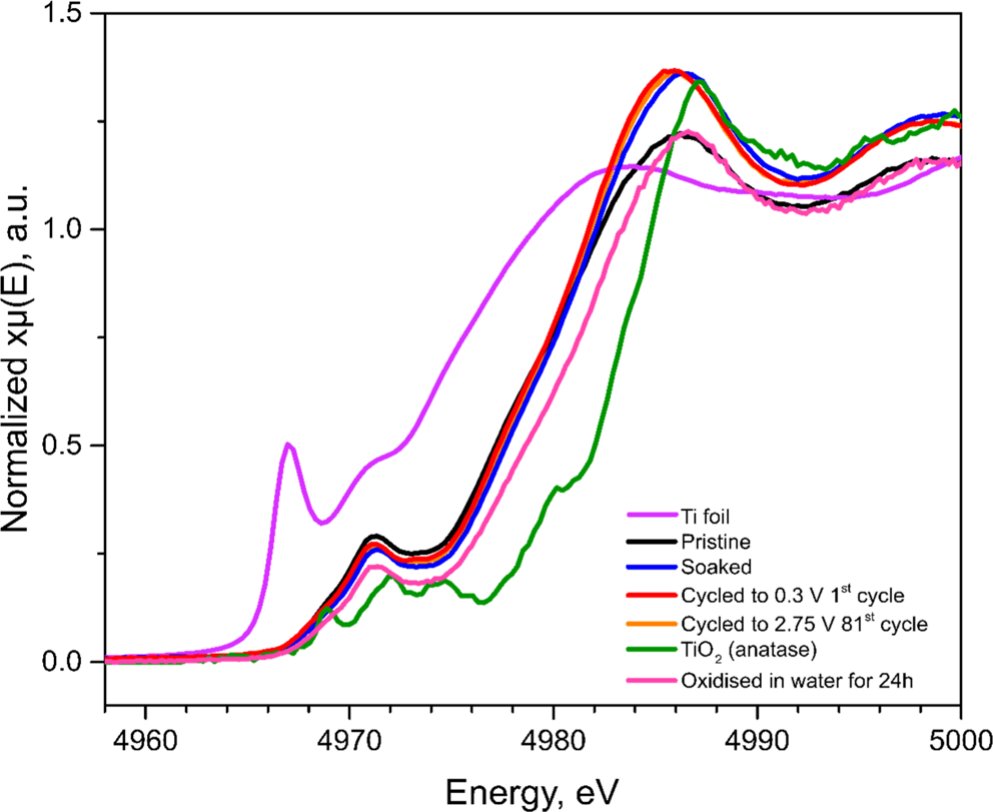
Ti K-edge XAS spectra obtained for differently treated Ti_3_C_2_*T*_*x*_ electrodes, that is, a pristine electrode (black), a Ti_3_C_2_*T*_*x*_ electrode soaked (but not cycled) in the electrolyte (blue), an electrode cycled to 0.3 V vs Li^+^/Li on the first cycle (red), an electrode cycled to 2.75 V vs Li^+^/Li on the 81st cycle (orange), and an electrode exposed to water and air for 24 h at room temperature (pink). The spectra for a titanium foil (purple) and TiO_2_ anatase (green) have been included for comparison.

To investigate if the obtained capacities stemmed from redox reactions involving the *T*_*x*_–Ti–C titanium species, experiments with bulk sensitive X-ray absorption spectroscopy (XAS) as well as surface sensitive X-ray photoelectron spectroscopy were performed. To minimize the change in the surface concentrations prior to the measurements (due to, e.g., self-discharge), the electrodes were transferred into the XPS instrument (or HAXPES end-station) without exposure to air using an argon-filled load-lock. The evaluation of the data was also based on the comparisons of the changes seen in the XPS results and in the electrochemical data for the different electrodes discussed below. As the same approach was used for all the different electrodes, comparisons of the results for the electrodes could still be used even though there may have been some changes in the oxidation states of the *T*_*x*_–Ti–C titanium species prior to the XPS measurements. It should also be noted that the rate of self-discharge would be expected to be lower for a delithiated (i.e., oxidized) electrode than that for a lithiated electrode as the concentration of species able to undergo oxidation at potentials up to about 3 V versus Li^+^/Li should be low in the electrolyte. To study the change in the titanium oxidation state within the electrode material, ex situ XAS of the Ti K-edge was performed in the transmission mode on pristine and cycled freestanding Ti_3_C_2_*T*_*x*_ electrodes. The XAS data (which should be less sensitive to self-discharge effects as XAS is a more bulk sensitive technique compared to XPS) for the pristine MXene are shown in Figure 3, together with the reference spectra for a titanium metal foil (for which the Ti oxidation state should be zero) and TiO_2_ anatase powder (for which the Ti oxidation state should be +IV). The titanium metal foil was used as a reference as the oxidation state of the Ti–C titanium present in the center of the MXene flakes also should be zero (see the Supporting Information). TiO_2_ was used as the other reference as the oxidation of the Ti_3_C_2_*T*_*x*_ MXene flakes by oxygen and/or water eventually should yield TiO_2_ and carbon (see the Supporting Information).^15,18,21^ As the oxidation of titanium to TiO_2_ is a four-electron oxidation reaction, one would, however, also expect to see intermediate titanium oxidation states such as Ti(II) and Ti(III) for the *T*_*x*_–Ti–C titanium present on the surfaces of the Ti_3_C_2_*T*_*x*_ flakes. In Figure 3, it is seen that the XAS spectrum of the pristine Ti_3_C_2_*T*_*x*_ electrode differed substantially from both reference spectra. In the pristine Ti_3_C_2_*T*_*x*_ XAS spectrum, it was thus not possible to identify any contributions from the three characteristic pre-edge peaks seen in the TiO_2_ XAS spectrum. Moreover, the positions of the edges were also substantially different for the reference spectra and the pristine Ti_3_C_2_*T*_*x*_ electrode. As the energy position of the XAS edge generally is correlated to the oxidation state of the element, the Ti K-edge positions for the Ti_3_C_2_*T*_*x*_ electrodes were determined from the maximum of the first derivative of normalized intensity with respect to incident energy (see Table S2). This indicated that the (average) oxidation state of the titanium in the Ti_3_C_2_*T*_*x*_ MXene electrodes was higher than zero (i.e., higher than that for the titanium foil) but lower than +IV (i.e., lower than that for the TiO_2_ reference sample). As the position of the Ti K-edge was similar for all samples (i.e., the pristine and the cycled electrodes), it can also be concluded that there was no significant change in the (average) Ti oxidation state during the electrochemical cycling. The only sample that showed a considerable Ti K-edge shift (of +1.5 eV compared to that for the pristine Ti_3_C_2_*T*_*x*_ electrode) was a freestanding electrode that had been left in a stirred open beaker containing deionized water for 24 h at room temperature.

The XAS results hence indicate that the (average) titanium oxidation state in the pristine Ti_3_C_2_*T*_*x*_ electrode was higher than zero but lower than +IV and that the main part of titanium was redox-inactive during the cycling of the electrodes. This is in good agreement with the low capacities seen during the cycling of the pristine MXene-based electrodes (see Figure 1). As is discussed in more detail in the Supporting Information, these findings support the hypothesis that the *T*_*x*_–Ti–C surface species on the MXene flakes in the pristine electrode contained oxidized titanium (e.g. Ti(II), Ti(III), and/or Ti(IV)). The XAS data also indicate that the pristine electrode did not contain substantial amounts of TiO_2_, that is, the expected surface oxidation to TiO_2_ and carbon was far from complete. The latter could be explained by the actions taken to minimize the exposure of the pristine sample to air and water, as well as the difficulties associated with the oxidation of each individual flake, particularly when there is restacking of the flakes. This slow oxidation hypothesis is further supported by the positive shift in the Ti K-edge (indicating an increase in the Ti oxidation state) seen for the electrode exposed to water and air for 24 h.

**Figure 4:**
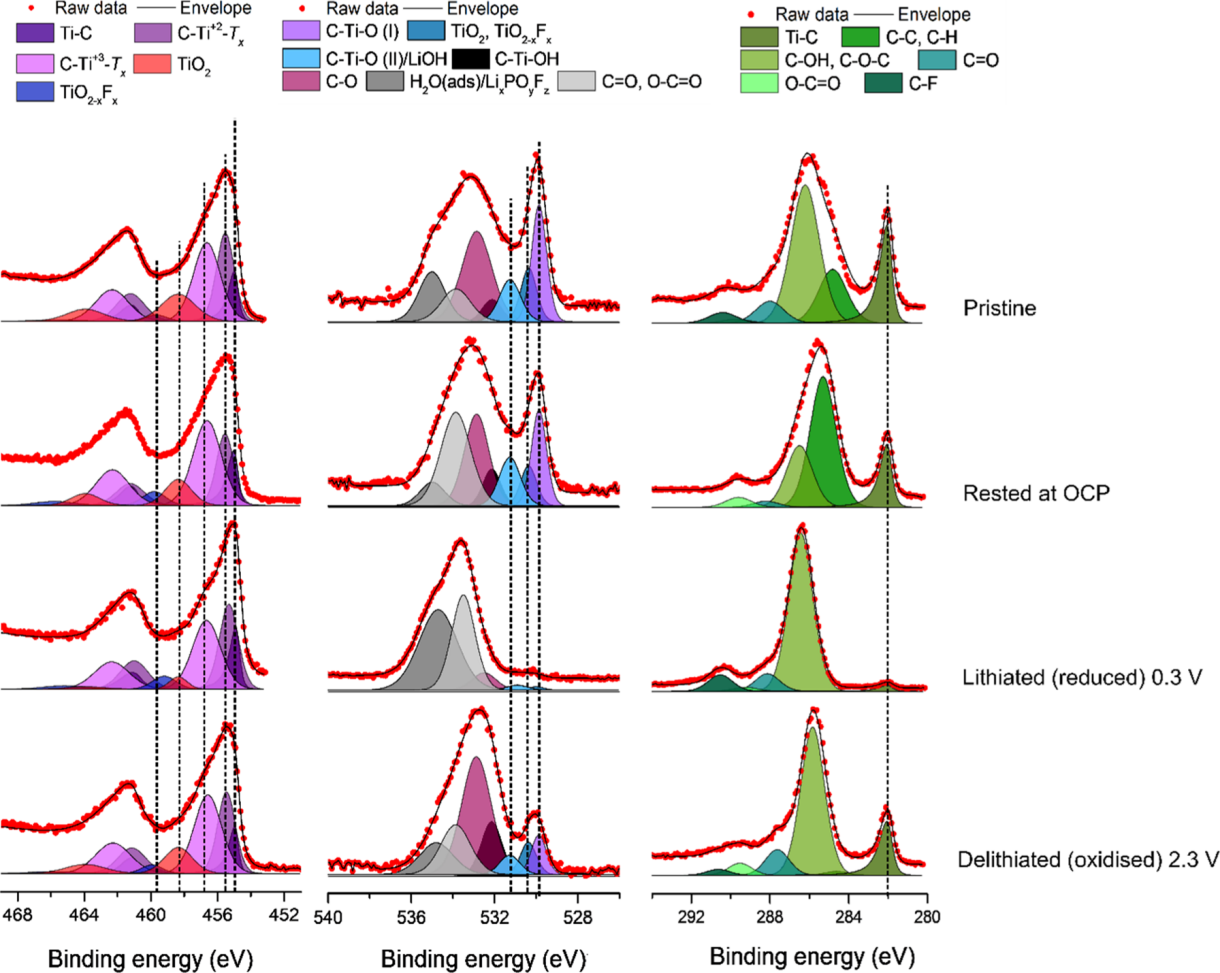
Ex situ XPS Ti 2p (left), O 1s (middle), and C 1s (right) spectra for a pristine Ti_3_C_2_*T*_*x*_ MXene electrode, an electrode exposed to the electrolyte under open-circuit conditions, an electrode lithiated (i.e., reduced) to 0.3 V vs Li^+^/Li on the first cycle as well as an electrode delithiated (i.e., oxidized) to 2.3 V vs Li^+^/Li on the first cycle, respectively.

The surfaces of the Ti_3_C_2_*T*_*x*_ pristine and cycled electrodes were also studied using XPS and HAXPES, as can be seen in Figures 4 and S4, respectively. The Ti 2p XPS spectra for the electrodes were deconvoluted (see Tables S3 and S4 in the Supporting Information) based on the reference data for Ti MXene species (i.e., Ti–C and Ti bonded to the surface termination groups such as −OH, =O, and −F) as well as TiO_2_ surface oxide.^18,23^ All Ti 2p spectra showed asymmetric peak shapes, indicating that the metallic type of bond was predominant. For each sample, the peaks assigned to the *T*_*x*_–Ti–C titanium surface species had higher relative intensities than those for the Ti–C component in the XPS spectra compared to that in the HAXPES spectra (compare Figures 4 and S4). This is not surprising as the spectra measured with lower photon energies (i.e., XPS) generally show relatively more of the surface components compared to measurements using higher photon energies (i.e., HAXPES). The results, hence, indicate that the Ti species containing Ti(II), Ti(III), and Ti(IV) were located closer to the surface than the Ti–C species. The surface species therefore most likely included titanium surface species such as C–Ti–O, C–Ti–OH, and C–Ti–F despite the fact that these Ti 2p peaks have been assigned to the Ti–C environment in some publications.^15,19,24^ The O 1s spectra were deconvoluted using seven different peaks. Going from lower to higher binding energies, the three first O 1s peaks were assigned to two C–Ti–O species, denoted C–Ti–O(I) and C–Ti–O(II)/LiOH as well as the TiO_2_ and TiO_2–*x*_F_*x*_ peaks.^23,25^ These peaks are followed by the C–Ti–OH peak at ∼532.1 eV and the peaks assigned to organic species: C–O, C=O, and O–C=O, adsorbed on the surface or as a part of the organic SEI. Lastly, the two peaks at even higher binding energies could be explained by the presence of weakly adsorbed water on the surface as well as inorganic fluorinated SEI components, for example, Li_*x*_PO_*y*_F_*z*_.^26,27^ By comparing the relative intensities of the *T*_*x*_–Ti–C surface species to those of the Ti–C peak (see Table S3 in the Supporting Information), it is clear that the relative intensity of the *T*_*x*_–Ti–C surface species (containing Ti(II), Ti(III), and Ti(IV)) decreased when the electrode was lithiated (i.e., reduced). This indicates that the lithiation resulted in a reduction of the *T*_*x*_–Ti–C titanium surface species. Note also that the relative intensities of the *T*_*x*_–Ti–C surface species increased during the subsequent delithiation (i.e., oxidation) step. These changes in the spectra support the hypothesis that the observed capacity (see Figure 1) stemmed from redox reactions involving *T*_*x*_–Ti–C titanium surface species. In the carbon spectra, the bulk carbon peak (due to Ti–C) was found at the lowest binding energy. At higher binding energies, carbon bonded to oxygen was found, indicating the presence of oxidized carbon species. Such species are, however, commonly found in battery electrodes and battery electrolytes.^28^

The main differences between the spectra for the electrodes cycled to different potentials included (i) a change in the Ti 2p intensity ratio between the *T*_*x*_–Ti–C surface species and Ti–C (see Table S3 in the Supporting Information), (ii) a change in the C 1s intensity ratio between the bulk Ti–C component and the surface components, and (iii) a change in the O 1s intensity ratio between the C–Ti–O(I) peak and the surface oxygen peak components. The changes in the C 1s and O 1s spectra (see the peak fitting results in Tables S5 and S6 in the Supporting Information) were mainly ascribed to the reduction of the electrolyte during the lithiation, resulting in the formation of a SEI layer on the electrode surface. This layer was to a large extent lost upon subsequent delithiation, most likely due to SEI dissolution.^29,30^ As a stable SEI was not formed on the MXene electrodes, additional SEI was consequently formed on each cycle. An estimation of the current due to the SEI formation process (see the Supporting Information), however, suggested that this current should have been of minor importance compared to that due to the reduction of the oxidized titanium surface species. The changes observed in the Ti 2p spectra, on the other hand, indicated the presence of redox reactions involving the *T*_*x*_–Ti–C surface species (containing Ti(II), Ti(III), and Ti(IV)). The relative ratios between the main peaks in Ti 2p, O 1s, and C 1s are summarized in Table S3 in the Supporting Information. The C–Ti–O(I) [O 1s]/Ti–C [Ti 2p] ratios were equal to 0.67, <0.01, and 0.76 for the soaked pristine, reduced (i.e., lithiated), and oxidized (i.e., delithiated) electrode, respectively. As a higher ratio was found for the delithiated (i.e., oxidized) electrode compared to both the soaked pristine and the reduced electrode, it is reasonable to assume that the *T*_*x*_–Ti–C surface species on the MXene flakes were redox-active, whereas Ti–C in the center of the flakes was not (the ratio Ti–C [C 1s]/Ti–C [Ti 2p] is relatively stable). The XPS/HAXPES data, therefore, support the conclusion based on the XAS data that a significant part of Ti was inactive during the cycling and that the redox activity was due to the presence of oxidized titanium containing surface species. This result thus indicates that the reversible capacity found for these Ti_3_C_2_*T*_*x*_ MXene electrodes (see Figure 1) stemmed from the redox activity of the *T*_*x*_–Ti–C surface species present on the MXene flakes (see Figure 2), whereas Ti–C present in the center of the flakes should have remained electro-inactive.

### Influence of the Degree of Surface Oxidation on the Electrochemical Performance

It is well known that the exposure of Ti_3_C_2_*T*_*x*_ to oxygen and/or water results in the oxidation of titanium, eventually yielding TiO_2_ anatase and carbon.^15,18,21^ The surfaces of the generated carbon can then also be oxidized by oxygen to give oxygen-containing surface groups (i.e., oxidized carbon surface species). While the exposure of the Ti_3_C_2_*T*_*x*_ material to air and water generally is minimized to prevent this oxidative degradation of the material, the results discussed above indicate that higher capacities should in fact be obtained when the *T*_*x*_–Ti–C surface species have been formed on the surface of the Ti_3_C_2_*T*_*x*_ MXene electrodes. As the oxidation of Ti to TiO_2_ is a four-electron process, the *T*_*x*_–Ti–C species may then include a mixture of Ti(II), Ti(III), and Ti(IV) species depending on the oxidation conditions. For a sufficiently long oxidation time, TiO_2_ should, however, mainly be seen. This means that the capacity of the electrode should depend on the degree of oxidation of the *T*_*x*_–Ti–C species present on the surfaces of the MXene flakes. The highest capacity should then be obtained with Ti(IV) species such as TiO_2_ present on the surfaces of the MXene flakes. Experiments were therefore designed to assess this hypothesis.

**Figure 5:**
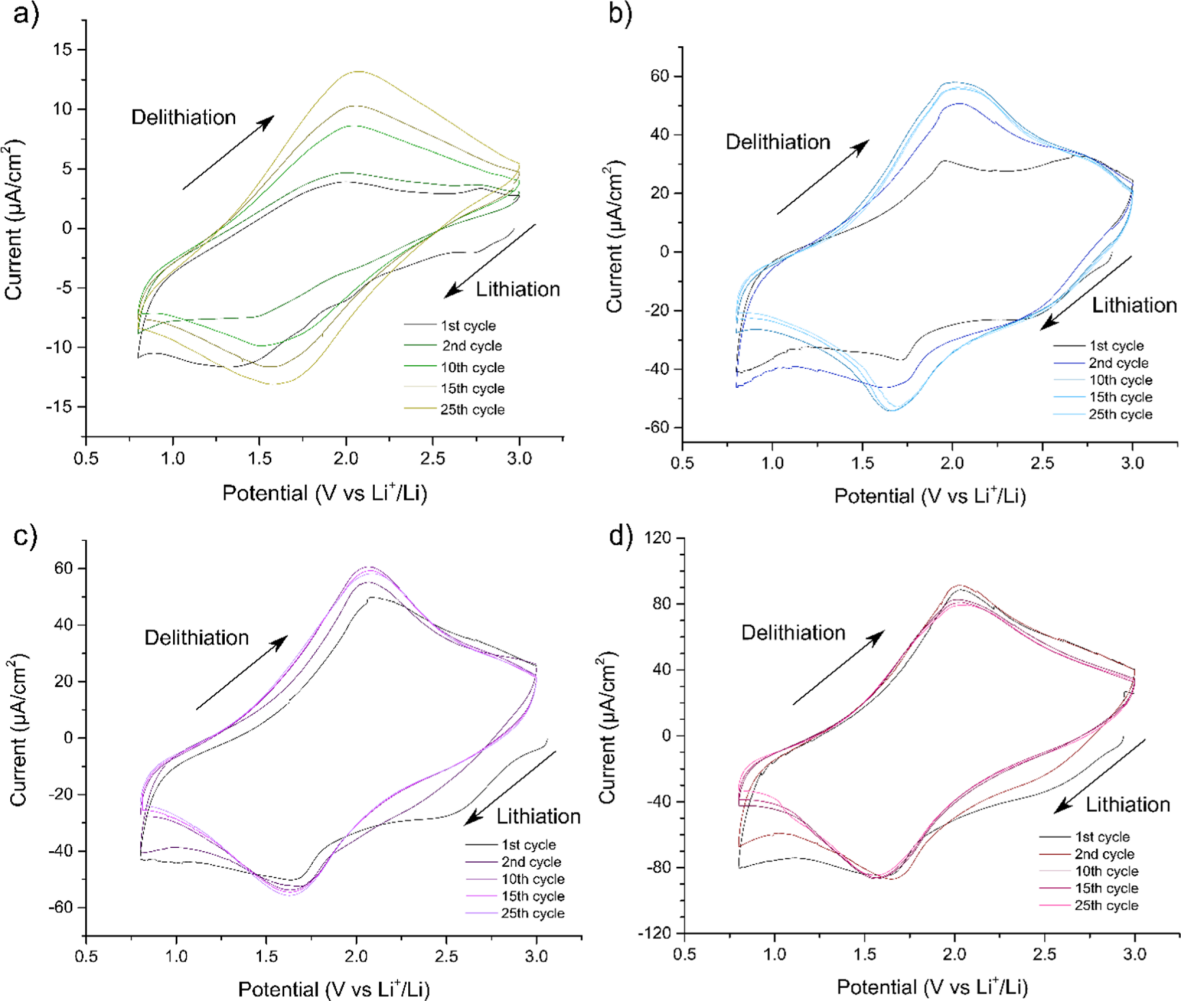
Cyclic voltammograms recorded at a scan rate of 0.1 mV s^–1^ for (a) pristine freestanding Ti_3_C_2_*T*_*x*_ film electrode, as well as the corresponding electrodes made from a suspension of Ti_3_C_2_*T*_*x*_ in deionized water exposed to air in an open vial for (b) 7, (c) 14, and (d) 28 days, respectively. The electrode mass loading in (a) was 1.31 mg.

To investigate the influence of the degree of oxidation of the Ti_3_C_2_*T*_*x*_ electrodes on their capacities, a suspension of the Ti_3_C_2_*T*_*x*_ MXene in water was kept in an open vial (i.e., in contact with air) during a period of 28 days. On the 7th, 14^th^, and 28th days of the experiment, 5 mL of the suspension with a concentration of between 3 and 4 mg/mL was taken from the vial with a syringe and vacuum-filtrated to obtain a freestanding film electrode which was then subjected to voltammetric cycling (see Figure 5). In addition to the abovementioned electrodes, a pristine electrode was also studied. Unlike that in Figure 1, a low cutoff limit of 0.8 V versus Li^+^/Li was used to avoid complications due to the potential conversion reaction involving any TiO_2_ formed on the surface of the electrode (i.e., TiO_2_ + 4Li^+^ + 4e^–^ = Ti + 2Li_2_O). As is explained in the Supporting Information, this conversion reaction would be expected to have a standard potential of about 0.6 V versus Li^+^/Li. As seen in Figure 5, all the obtained voltammograms featured a lithiation (i.e., reduction) peak at about 1.7 V versus Li^+^/Li and a delithiation (i.e., oxidation) peak at about 2 V versus Li^+^/Li. As this is in very good agreement with the results seen in Figure 1b, it is reasonable to assume that there was no significant influence of the abovementioned conversion reaction on the electrochemical performance of the electrode. More importantly, the results in Figure 5 clearly show that the lithiation (i.e., reduction) and delithiation (i.e., oxidation) peak currents increased with the number of days during which the Ti_3_C_2_*T*_*x*_ material was exposed to water and air in the open vial. The capacity of the Ti_3_C_2_*T*_*x*_ electrode thus increased when the surfaces of the Ti_3_C_2_*T*_*x*_ flakes were oxidized. Here, it should also be noted that the potentials of the reduction and oxidation peaks are in good agreement with those generally seen for the lithiation and delithiation of TiO_2_.^31,32^ It is therefore reasonable to assume that the increasing electrode capacity seen in Figure 5 was due to an increasing concentration of more oxidized titanium species, such as TiO_2_, on the surfaces of the Ti_3_C_2_*T*_*x*_ flakes.

**Figure 6:**
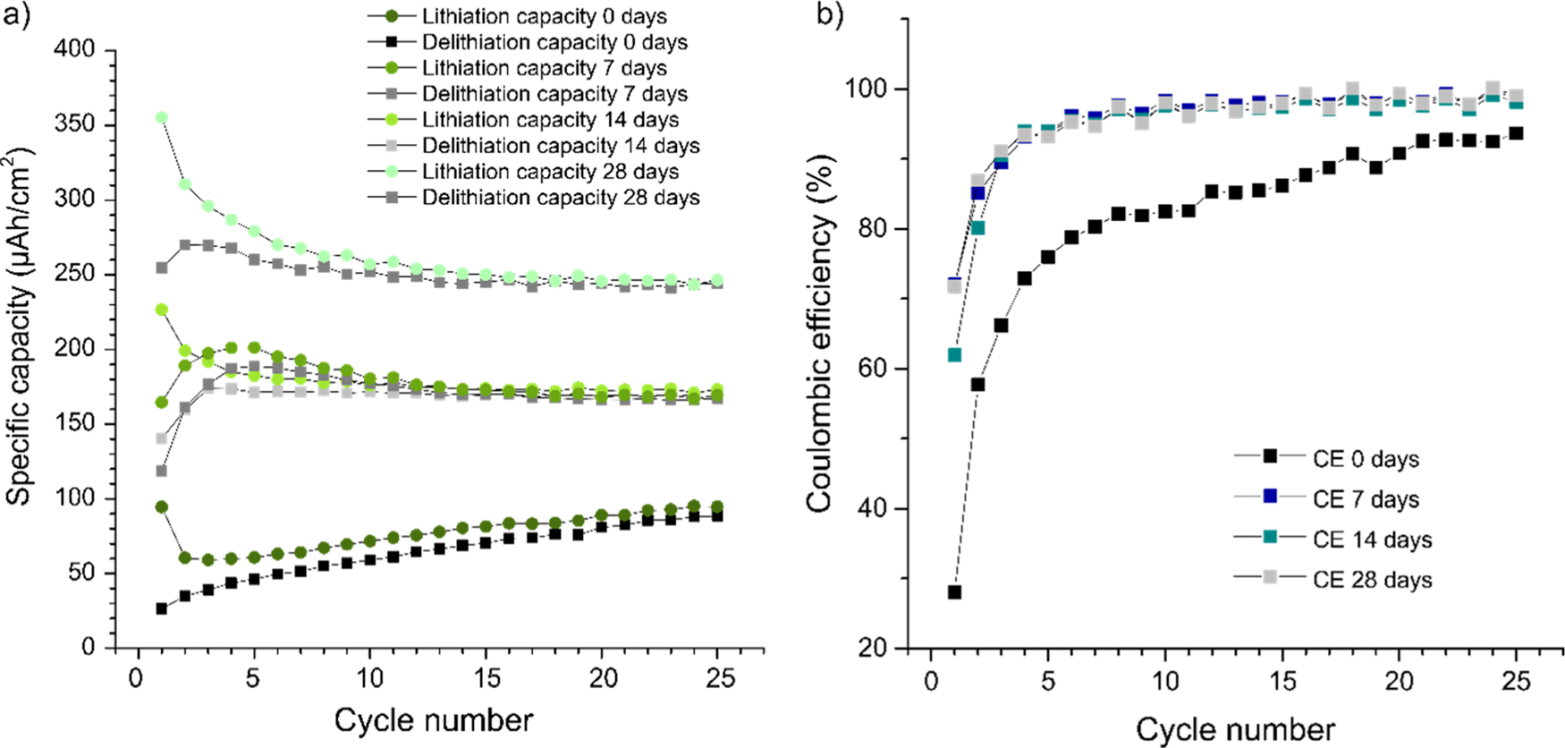
(a) Lithiation (i.e., reduction) and delithiation (i.e., oxidation) capacities, as well as the (b) associated Coulombic efficiencies, as a function of the cycle number. These values were evaluated from the voltammograms in Figure 5 for a pristine electrode and the electrodes prepared from a suspension of Ti_3_C_2_*T*_*x*_ in deionized water maintained in an open vial (i.e., in contact with air) for 7, 14, and 28 days, respectively.

The trends seen in the voltammetric data can be more clearly seen in the obtained specific capacities and Coulombic efficiencies, presented in Figure 6. The average capacity thus increased with the time the Ti_3_C_2_*T*_*x*_ suspension was exposed to water and air. For the pristine electrode and the electrode based on the Ti_3_C_2_*T*_*x*_ suspension exposed to water and air for 7 days, the capacities remained relatively constant, yielding about 21 and 50 mA h g^–1^ after 25 cycles, respectively. In the pristine electrode case, the lithiation and delithiation capacities, however, increased somewhat during the cycling, whereas the lithiation and delithiation capacities for the 7 day electrode reached a maximum (i.e., 60 and 57 mA h/g, respectively) on the fourth cycle. The lithiation capacities for the 14 and 28 day electrodes, on the other hand, decreased from 89 mA h g^–1^ on the first cycle to 68 mA h g^–1^ after 25 cycles for the 14 day electrode. The corresponding values for the 28 day electrode were 106 and 74 mA h g^–1^. These results thus indicate that the electrochemical performances of the Ti_3_C_2_*T*_*x*_ electrodes depended not only on their exposure to air and water but also on their cycling history. Still, when comparing the obtained capacities with that of about 165 mA h g^–1^ for the lithiation of anatase TiO_2_ to Li_*x*_TiO_2_, assuming *x* = 0.5,^33^ it is immediately clear that only a fraction of the MXene flakes could have been oxidized to TiO_2_. As it is reasonable to assume that there was some restacking of the Ti_3_C_2_*T*_*x*_ flakes after their manufacturing or during the manufacturing of the electrodes, this could have slowed down the oxidation rate of the MXene flakes to yield a lower electrode capacity. This could also explain why relatively long times (i.e., up to 28 days) were needed to oxidize the Ti_3_C_2_*T*_*x*_ suspension despite the fact that the suspension was in contact with both oxygen and water. The results hence indicate that while the surfaces of the Ti_3_C_2_*T*_*x*_ flakes underwent oxidation involving the formation of *T*_*x*_–Ti–C species containing oxidized titanium, for example, TiO_2_, the electrode still contained significant amounts of electro-inactive material even after an exposure to water and air for 28 days.

**Figure 7:**
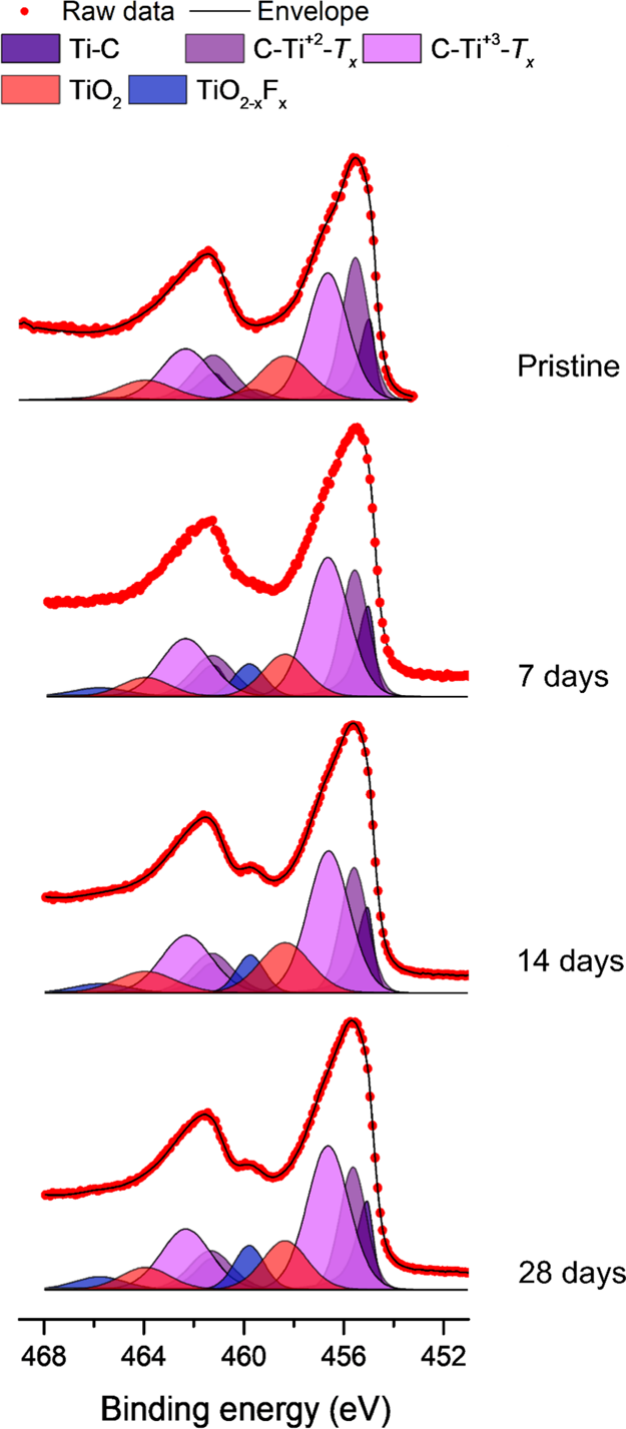
Ti 2p XPS spectra for a pristine freestanding Ti_3_C_2_*T*_*x*_ film as well as films prepared from an aqueous Ti_3_C_2_*T*_*x*_ suspension exposed to air for 7, 14, and 28 days, respectively. The corresponding C 1s and O 1s spectra are shown in Figure S5.

The surfaces of the electrodes used in the abovementioned oxidation experiment were also studied using XPS (see Figure 7). In this case, there should not have been any significant self-discharge prior to the XPS measurements due to the absence of a suitable reducing agent (as is explained in the Supporting Information, the oxidation of the Ti_3_C_2_*T*_*x*_ flakes is a spontaneous process, ultimately yielding TiO_2_). The analysis of the Ti 2p region clearly showed that a layer of both TiO_2_ and TiO_2–*x*_F_*x*_ was formed on the surface of the Ti_3_C_2_*T*_*x*_ flakes upon exposure to water and air. This was evident from the relative increase in intensity of the peaks TiO_2_ at 458.3 eV and TiO_2–*x*_F_*x*_ at 459.8 eV compared to that for the pristine Ti 2p when increasing the number of days in the open vial. The data consequently indicate that the surfaces of the MXene flakes were at least partially converted into TiO_2_ (TiO_2–*x*_F_*x*_) upon exposure to air and water. As it is well known that TiO_2_ can be used as a negative electrode material for lithium-ion batteries,^22,32,34^ the formation of TiO_2_ on the surface of the Ti_3_C_2_*T*_*x*_ flakes should increase the capacity of Ti_3_C_2_*T*_*x*_-based electrodes significantly. Such a formation of a layer of TiO_2_ on titanium carbide is also compatible with the previously obtained results.^12,17^ In conjunction with Figure 7, it is, however, important to note that the XPS data confirm that the pristine Ti_3_C_2_*T*_*x*_ contained very little TiO_2_ and TiO_2–*x*_F_*x*_, most likely as particular care was taken not to expose the Ti_3_C_2_*T*_*x*_ MXene to air (i.e., oxygen). The results hence indicate that the capacity of a pristine Ti_3_C_2_*T*_*x*_ MXene electrode should be too low to be of practical importance for use as a negative electrode in lithium-ion batteries.

### Influence of Water on the Irreversible Capacity

**Figure 8:**
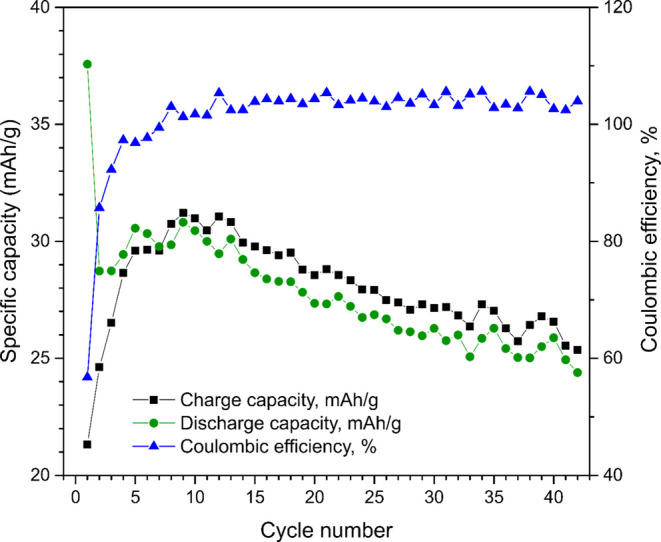
Lithiation (i.e., reduction) and delithiation (i.e., oxidation) capacities and Coulombic efficiency as a function of the cycle number for the galvanostatic cycling at 10 mA g^–1^ of a freestanding Ti_3_C_2_*T*_*x*_ electrode heat-treated for 16 h at 300 °C under vacuum (the cycling curves can be seen in Figure S6 in the Supporting Information).

**Figure 9:**
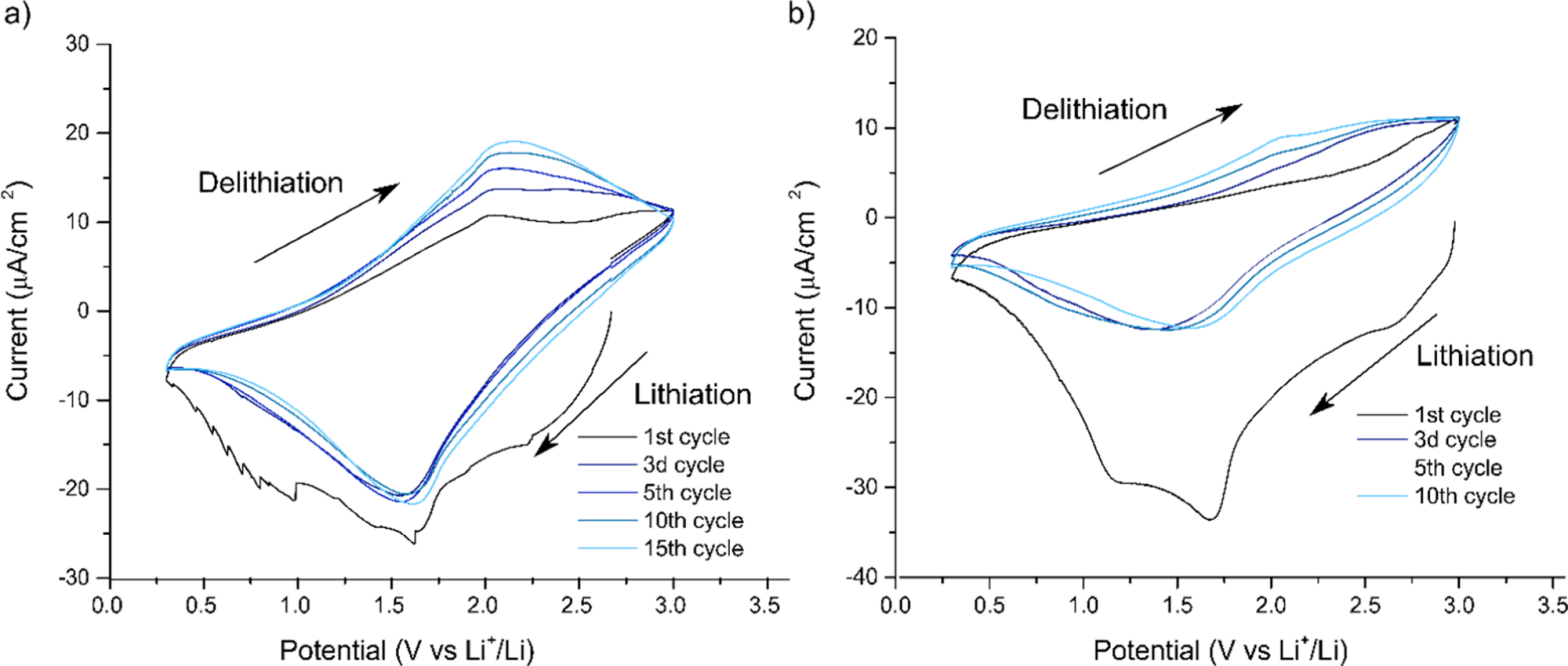
Cyclic voltammograms recorded between 0.3 and 3 V vs Li^+^/Li at a scan rate of 0.1 mV s^–1^ for a (a) freestanding Ti_3_C_2_*T*_*x*_ electrode heat-treated for 16 h at 300 °C under vacuum and a (b) nondried freestanding Ti_3_C_2_*T*_*x*_ electrode.

As can be seen in Figure 1, the first lithiation (i.e., reduction) capacity was much larger than both the corresponding oxidation capacity and the subsequent reduction capacities. This clearly indicates the presence of a significant irreversible capacity. What could this irreversible capacity be due to? There are at least two effects that should be considered here, that is, reduction of adsorbed water and SEI formation. While SEI formation should be seen at potentials below about 1 V versus Li^+^/Li, the onset of the reduction of adsorbed water should be seen at higher potentials, for example, 1.6 V versus Li^+^/Li.^35^ Water confinement between the MXene flakes is in fact a known phenomenon.^14,19,36^ It is also well known^31^ that the reduction of adsorbed water can give rise to large irreversible capacities for TiO_2_ electrodes and that it can be difficult to remove water completely (see Discussion in the Supporting Information). As there should be oxygen on the surfaces of the Ti_3_C_2_*T*_*x*_ flakes, it is reasonable to expect a similar behavior for the Ti_3_C_2_*T*_*x*_ electrodes used here. Experiments were therefore conducted involving constant current and voltammetric cycling of Ti_3_C_2_*T*_*x*_ electrodes, which either had been dried at 300 °C for 16 h in a vacuum or not at all (see Figures 8 and 9). While the irreversible capacity due to SEI formation (i.e., the reduction of the electrolyte), in principle, should be the same in both cases, a lower irreversible capacity due to the reduction of water should clearly be expected for the electrode dried at 300 °C (see also the Supporting Information).

A comparison of the voltammograms in Figure 9 clearly shows that a larger irreversible capacity was seen for the nondried electrode. It can also be seen that only a relatively small part of the irreversible capacity was due to reduction below 1 V versus Li^+^/Li (Figure 9). The latter suggests that the main part of the irreversible capacity was due to the reduction of adsorbed water. It should also be noted that all the voltammograms in Figures 8 and 9 except the first cycles feature a broad reduction peak at about 1.5–1.6 V vs Li^+^/Li as well as a broad oxidation peak at about 2 V versus Li^+^/Li in analogy with the voltammograms seen in Figures 1 and 5.

The performance of the electrode dried at 300 °C for 16 h (see Figures 8 and 9) can also be compared with that seen in Figure 1 for an electrode dried at 120 °C for 16 h, although it should be noted that different cycling windows were used in these two experiments. The lower first-cycle lithiation capacity seen for the electrode dried at 300 °C can be explained by a smaller contribution from the reduction of adsorbed water. The Coulombic efficiencies were also generally higher for the electrode dried at 300 °C for 16 h and reached ∼100% after less than 10 cycles (see also the cycling curves shown in Figure S6). The results therefore indicate that the irreversible capacity was smaller for the more extensively dried electrode. While the irreversible capacity on the first cycles could be due to both the reduction of adsorbed water and that of the electrolyte giving rise to an SEI layer, the results indicate that the largest effect was due to adsorbed water.

**Figure 10:**
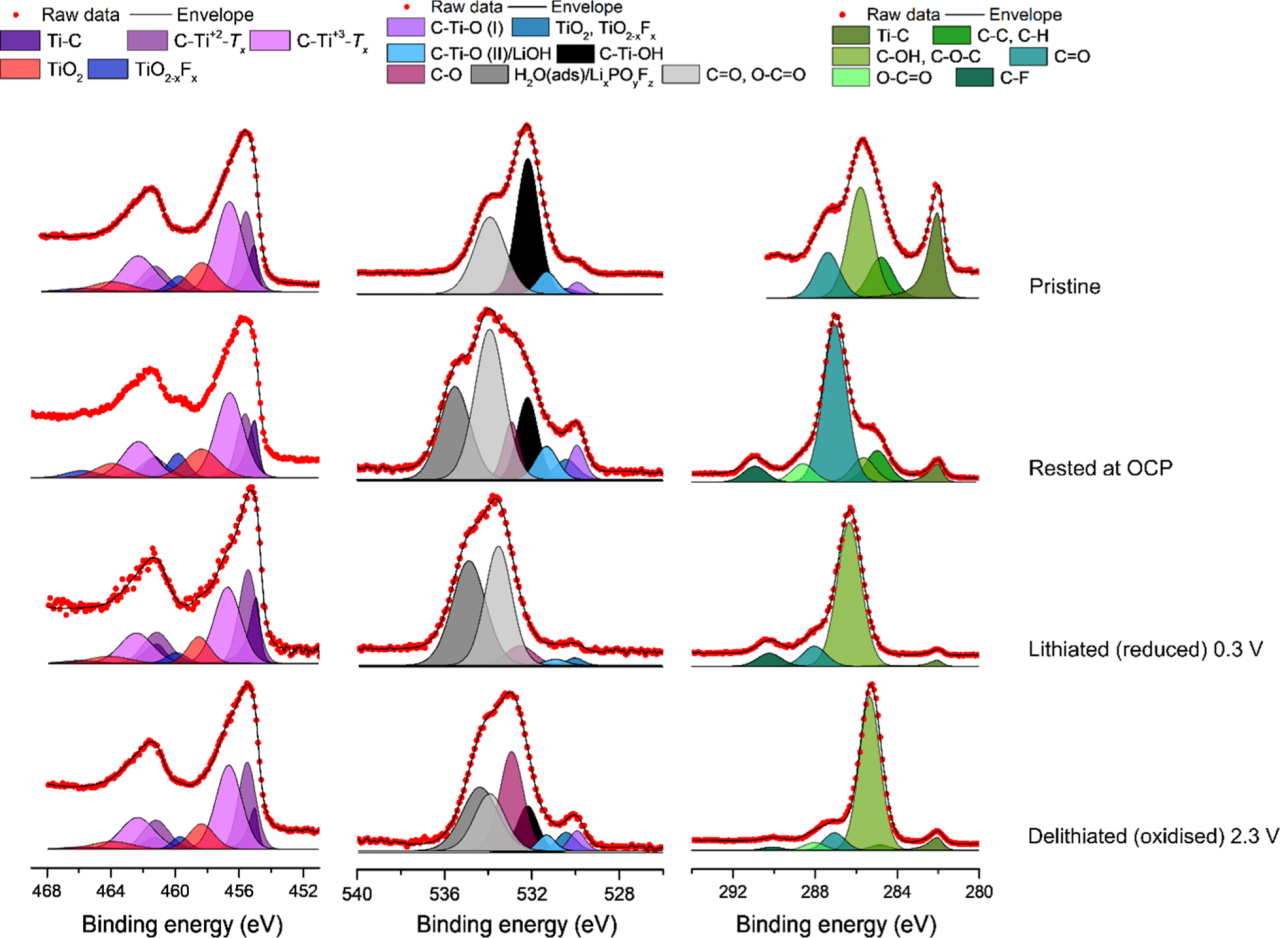
Ti 2p (left), O 1s (middle), and C 1s (right) XPS spectra for Ti_3_C_2_*T*_*x*_ electrodes dried at 300 °C for 16 h in a vacuum. The top spectra were obtained with a pristine sample, while the spectra below were obtained with a sample soaked in the electrolyte at the OCP. The two lower sets of spectra were obtained with an electrode that was first lithiated to 0.3 V vs Li^+^/Li and then delithiated to 2.3 V vs Li^+^/Li, respectively, on the first cycle.

To further study the effect of the drying step on the electrodes, XPS studies were made on three Ti_3_C_2_*T*_*x*_ electrodes dried at 300 °C for 16 h, where one electrode remained pristine, one was merely soaked in the electrolyte, while the third electrode was subjected to first-cycle lithiation (i.e., reduction) and delithiation (i.e., oxidation) to 0.3 and 2.3 V versus Li^+^/Li, respectively. The results for the pristine electrode (see Figure 10) show that the drying of the electrode at 300 °C only resulted in minor changes in the Ti 2p spectrum, seen as an increase in *T*_*x*_–Ti–C surface species relative to the Ti–C peak (compare Figures 4 and 10). Larger changes were, on the other hand, observed in the O 1s and C 1s regions. In the O 1s spectrum, the relative intensities of the C–Ti–OH and C=O, O–C=O peaks were substantially increased, while the H_2_O (ads) intensity decreased compared to the C–Ti–O(I) peak. This indicates that the drying at 300 °C resulted in a *T*_*x*_ termination group rearrangement, loss of water, and buildup of oxygen-rich surface species. At the same time, the C 1s spectrum showed a large increase of the C=O species and a small decrease of C–O–C and C–OH relative to the C 1s Ti–C peak as well as a probable depression of the C–F peak. It is therefore reasonable to assume that during the drying process at 300 °C under vacuum, water adsorbed on the surface of the Ti_3_C_2_*T*_*x*_ electrode reacted to yield titanium oxides as well as oxidized carbon and oxygen-containing species on the surface of the electrode. This is not unexpected as this reaction also should take place at room temperature, albeit at a lower rate.

For the soaked electrode (Figure 10), no major changes were seen in the Ti 2p spectrum compared to that for the pristine electrode. There was, however, a relative increase in C=O and O–C=O peaks in the O 1s spectrum and C=O peak in the C 1s spectrum. These changes were most likely caused by the adsorption of electrolyte degradation products present in the electrolyte due to the reaction of the electrolyte with the lithium metal electrode.

After lithiation (i.e., reduction) to 0.3 V versus Li^+^/Li on the first cycle, lower intensities were seen for the *T*_*x*_–Ti–C surface species (i.e., the Ti(II), Ti(III), and Ti(IV) species) compared to that for the pristine electrode, while the intensity of the Ti–C peak remained approximately the same. As decreased intensities likewise were seen for titanium containing oxygen and carbon species, the results demonstrate that the lithiation involved a reduction of *T*_*x*_–Ti–C species. From the O 1s and C 1s spectra, it can further be concluded that the lithiation step resulted in a thicker overlayer composed of C–O–C- and C–OH- containing species due to the formation of an SEI layer. After the subsequent delithiation (i.e., oxidation) to 2.3 V versus Li^+^/Li, the relative intensity of the *T*_*x*_–Ti–C surface species generally increased relative to that of the Ti–C peak in the Ti 2p region. A corresponding increase in the intensities for the *T*_*x*_–Ti–C species was seen in the O 1s region. An increase in the intensity of the TiO_2_ and TiO_2–*x*_F_*x*_ peaks in the Ti 2p spectrum was, however, not seen. This indicates that the extent of oxidation of titanium to Ti(IV) was limited during the delithiation step, most likely due to the formation of a surface layer of titanium species with lower oxidation states acting as a passivating layer. An analogous effect was previously described for SnO_2_ electrodes^37,38^ where a complete reformation of the initial SnO_2_ was very difficult to obtain during the delithiation step. Such problems yield lithiation (i.e., reduction) capacities which are significantly larger than the corresponding delithiation (i.e., oxidation) capacities during the initial part of cycling, in good agreement with the results presented above.

As is shown in Figure S7, XAS experiments were also conducted on the soaked, lithiated, and delithiated electrodes, complementary to the XPS experiments discussed above. The XAS results indicated that there was a small change in the e_g_/t_2g_ ratio. This suggests a small change in the titanium oxidation state at the surface (i.e., within a depth of a few nanometers) of the electrodes during the first cycle, in good agreement with the XPS results discussed above.

### What Determines the Reversible and Irreversible Capacity of the Ti_3_C_2_*T*_*x*_ Electrodes?

The results discussed above demonstrate that the *T*_*x*_–Ti–C species present on the surfaces of the Ti_3_C_2_*T*_*x*_ flakes give rise to the observed reversible capacity. These *T*_*x*_–Ti–C species contain oxidized titanium (i.e., Ti(II), Ti(III), and/or Ti(IV) species) that can undergo reduction during the lithiation step and oxidation during the subsequent delithiation step. The shapes of the cyclic voltammograms and chronopotentiograms, as well as the Nyquist plots shown in Figure S8, further indicate that the different redox reactions overlapped during the reduction and oxidation steps. Redox-active *T*_*x*_–Ti–C surface species should in fact have been formed already during the manufacturing of the MXene flakes as a result of the following redox reaction: Ti_3_C_2_ + 2H_2_O = Ti_3_C_2_(OH)_2_ + H_2_.^2^ In this reaction, water is clearly reduced to yield hydrogen. This means that there must be an accompanying oxidation involving either Ti or C. As Ti is easier to oxidize than carbon, Ti(II)-, Ti(III)-, and/or Ti(IV)-containing *T*_*x*_–Ti–C species are formed on the surface of the MXene flakes, while the center of the flakes still contains titanium only bonded to carbon (i.e., the Ti–C titanium). The titanium bonded to Cl, F, and O on the surface of the MXene flakes should thus have an oxidation state higher than zero, whereas the titanium present in Ti_3_C_2_, as well as in Ti–C in the center of the Ti_3_C_2_*T*_*x*_ flakes, should be elemental. The oxidation state of the titanium in the *T*_*x*_–Ti–C surface species should, however, also depend on the time the MXene flakes are exposed to oxygen, water, or other oxidizing species. This explains the increase in the capacity seen when exposing the Ti_3_C_2_*T*_*x*_ flakes to water and oxygen for increasing times discussed above. The fact that the *T*_*x*_–Ti–C surface species would be oxidized to different degrees during the manufacturing and electrode preparation procedures is most likely one important reason for the large variation in the reversible capacities reported when Ti_3_C_2_*T*_*x*_ is used as a negative electrode material for lithium-ion batteries.

The capacities of Ti_3_C_2_*T*_*x*_ MXene electrodes will clearly also depend on the extent of restacking of the MXene flakes, as this will hinder the oxidation of the surfaces of the MXene flakes. When using Ti_3_C_2_*T*_*x*_ as a negative electrode material for lithium-ion batteries, it is, therefore, essential to make sure that all MXene flakes remain in contact with the electrolyte. To obtain a high degree of oxidation of the Ti_3_C_2_*T*_*x*_ material, the surface area of the material clearly needs to be maintained as large as possible. The ideal Ti_3_C_2_*T*_*x*_ electrode should therefore contain a large number of individual Ti_3_C_2_*T*_*x*_ MXene flakes, where each flake should have a Ti–C core coated with *T*_*x*_–Ti–C surface species, according to the schematic illustration in Figure 2. In this context, it should also be pointed out that it would be more appropriate to refer to the redox reactions involving the *T*_*x*_–Ti–C surface species as surface-confined redox reactions rather than conventional intercalation reactions.

The results further suggest that the previously predicted reduction of the elemental carbon present in Ti_3_C_2_, Ti_3_C_2_F_2_, or Ti_3_C_2_(OH)_2_ to yield Ti_3_C_2_Li_2_, Ti_3_C_2_F_2_Li_2_, or Ti_3_C_2_(OH)_2_Li_2_ (e.g., Ti_3_C_2_(OH)_2_ + 2e^–^ + 2Li^+^ = Ti_3_C_2_(OH_2_)Li_2_) do not appear to take place at potentials above 0 V versus Li^+^/Li, despite the fact that their standard potentials have been calculated^13^ to be 0.62, 0.56, and 0.14 V versus Li^+^/Li, respectively. As the titanium in Ti_3_C_2_ likewise is elemental, this means that Ti_3_C_2_ should be electrochemically inactive (see the discussion in the Supporting Information) during the lithiation step. It is therefore inappropriate to use the theoretical capacities (i.e., 320, 130, and 67 mA h g^–1^, respectively) for the abovementioned reactions in conjunction with the use of Ti_3_C_2_*T*_*x*_ as a negative lithium-ion electrode material.

Even though the capacity of the Ti_3_C_2_*T*_*x*_ electrodes mainly stems from the presence of oxidized *T*_*x*_–Ti–C species, it is still very difficult to fully identify the redox reactions involved. The reason for this is that there can be many different oxidized titanium species yielding overlapping redox reactions (see the list of possible redox reactions in the Supporting Information). Some examples of possible redox reactions can be found in the Supporting Information. It is, however, clear that the full oxidation of the *T*_*x*_–Ti–C surface species should result in the formation of TiO_2_ and carbon. This means that the capacities of Ti_3_C_2_*T*_*x*_ electrodes should approach those of the corresponding TiO_2_ electrodes. Here, it should be noted that the capacity for a TiO_2_ electrode typically is measured between about 1.2 and 3 V versus Li^+^/Li rather than between 0 and 3 V versus Li^+^/Li as the redox reaction is only assumed to involve a reduction of Ti(IV) to Ti(III) according to TiO_2_ + *x*e^–^ + *x*Li^+^ = Li_*x*_TiO_2_. As *x* typically is equal to about 0.5, the attainable capacity for a TiO_2_ electrode is then typically around 170 mA h/g.^31,32^ Higher capacities should, however, be seen when cycling to lower potentials than 1.2 V versus Li^+^/Li. The capacity contribution due to the presence of oxidized carbon surface species is more difficult to estimate as these typically yield a pseudocapacitive behavior rather than peaks that can be seen in the voltammograms. The present results, nevertheless, indicate that the main capacity of the Ti_3_C_2_*T*_*x*_ electrodes stemmed from redox reactions involving oxidized titanium surface species. While this is in good agreement with the findings for oxidized Ti_3_C_2_*T*_*x*_ electrodes,^15,16,18^ the general recommendation, however, still appears to be to avoid the oxidation of the Ti_3_C_2_*T*_*x*_ flakes as much as possible. One reason for the latter recommendation is that the full oxidation to TiO_2_ and carbon typically results in the disintegration of the electrode material.^18,39^

A large part of the irreversible capacity seen on the initial cycles most likely stemmed from the reduction of water adsorbed on the surface of the Ti_3_C_2_*T*_*x*_ material, in analogy with the findings for TiO_2_ electrodes.^31^ As the present results show that it is very difficult to remove this water, great care should be taken when drying the material prior to its use in nonaqueous batteries. The fact that adsorbed water most likely will contribute to the lithiation capacity during the initial part of the cycling should naturally be considered when reporting the capacities for the material. The present results indicate that the irreversible capacity contribution from the formation of the SEI typically is less important than that due to the reduction of the adsorbed water. While an incomplete oxidation to TiO_2_ during the delithiation (i.e., oxidation) step likewise can contribute to the irreversible capacity as indicated by the XPS results, it is difficult to quantify this effect mainly due to the large influence from the reduction of the adsorbed water.

## Conclusions

The present results demonstrate that the reversible capacity seen for freestanding Ti_3_C_2_*T*_*x*_ MXene films, when used as negative electrodes in lithium-ion batteries, mainly stems from the presence of oxidized titanium and carbon species on the surfaces of the Ti_3_C_2_*T*_*x*_ MXene flakes. Spontaneous oxidation of the flakes due to contact with air and/or water was demonstrated to result in an increased concentration of oxidized surface species, yielding an increased reversible capacity. The previously suggested reduction of the elemental carbon in Ti_3_C_2_*T*_*x*_, predicted to take place below about 0.6 V versus Li^+^/Li, could not be verified experimentally. As the Ti–C titanium, present in the center of the Ti_3_C_2_*T*_*x*_ flakes, should be elemental, this titanium would not be expected to contribute to the lithiation (i.e., reduction) capacity at potentials between 3 and 0 V versus Li^+^/Li. This is in excellent agreement with the obtained electrochemical, XPS, HAXPES, and XAS results. As a result, pristine Ti_3_C_2_ is consequently not a promising negative electrode material for lithium-ion batteries. The capacity of electrodes composed of Ti_3_C_2_*T*_*x*_ MXene flakes hence depends on the oxidation state of the titanium present in the *T*_*x*_–Ti–C surface species. In contrast to the general recommendations to minimize the contact between the Ti_3_C_2_*T*_*x*_ MXene and oxygen and water, the attainment of a high degree of oxidation of the *T*_*x*_–Ti–C surface species is essential when using Ti_3_C_2_*T*_*x*_ MXene-based negative electrodes for lithium-ion batteries.

The significant irreversible capacity seen for the Ti_3_C_2_*T*_*x*_ MXene electrodes during the initial cycles can mainly be ascribed to the reduction of adsorbed water, although there should also be contributions from SEI formation and the inability to fully reform the oxidized titanium surface species on the delithiation (i.e., oxidation) steps. To eliminate the influence of the residual water, more attention should be paid to drying of the electrodes prior to their use in lithium-ion batteries. The present results show that water was still present in the Ti_3_C_2_*T*_*x*_ electrodes even after drying for 16 h at 300 °C in a vacuum.

As oxidized titanium and carbon surface species will be formed gradually on the surface of the Ti_3_C_2_*T*_*x*_ flakes when these are exposed to air and/or water, the reversible capacity of Ti_3_C_2_*T*_*x*_ electrodes should depend on the exposure time and the experimental conditions employed. Since the Ti–C, situated underneath the *T*_*x*_–Ti–C surface layer, is electrochemically inactive (see the discussion in the Supporting Information), the capacity of Ti_3_C_2_*T*_*x*_ electrodes should depend on the degree of oxidation of the *T*_*x*_–Ti–C surface layer, the porosity of the electrode, as well as the degree of restacking of the Ti_3_C_2_*T*_*x*_ MXene flakes in the electrode. This means that the capacity of Ti_3_C_2_*T*_*x*_ MXene-based electrodes should depend on the procedures used to manufacture, wash, store, and dry the obtained material prior to its use as a negative electrode material in lithium-ion batteries. This can explain the significant variation in the reversible capacities seen in the literature. As the full oxidation of the titanium in Ti_3_C_2_*T*_*x*_ should result in the formation of TiO_2_ (and carbon), the reversible capacities for such Ti_3_C_2_*T*_*x*_ electrodes should be comparable to those obtained for TiO_2_ electrodes. When using Ti_3_C_2_*T*_*x*_ as a negative electrode material in lithium-ion batteries, Ti_3_C_2_*T*_*x*_ should consequently be subjected to a pretreatment step, resulting in the formation of Ti(II), Ti(III), and Ti(IV) surface species.

## Supporting Information Available

Additional information regarding possible redox reactions involving Ti_3_C_2_*T*_*x*_ electrodes; average oxidation state of the Ti_3_C_2_*T*_*x*_ electrodes and the estimated capacities due to double-layer charging; SEI formation and reduction of adsorbed water; cross-sectional SEM image of a Ti_3_C_2_*T*_*x*_ electrode; additional cycling curves and data of galvanostatic as well as voltammetric lithiation and delithiation capacities; Nyquist plots recorded at different potentials; HAXPES and XPS spectra; XAS spectra; XPS main peak ratios; and details regarding the XPS peak-fitting routines (PDF)
