# Supplementary material for: On the Use of Ti3C2Tx MXene as a Negative Electrode Material for Lithium-Ion Batteries
Source: ACS Omega. 2022 Nov 7;7(45):41696–710. doi: 10.1021/acsomega.2c05785 (PMC9670687; doi:10.1021/acsomega.2c05785)
Supplement: Supplementary file 1 — ao2c05785_si_001.pdf [file ao2c05785_si_001.pdf]

Supporting Information

On the use of $Ti_3C_2T_x$ MXene as a negative electrode material for lithium-ion batteries

Tatiana Koriukina[a], Antonia Kotronia[a], Joseph Halim[b], Maria Hahlin[a], Johanna Rosen[b], Kristina Edström[a] and Leif Nyholm[a]*

[a]The Ångström Advanced Battery Center, Department of Chemistry–Ångström Laboratory, Uppsala University, Box 538, SE-751 21 Uppsala, Sweden

[b]Materials Design Division, Department of Physics, Chemistry and Biology (IFM), Linköping University 58183 Linköping, Sweden

1. Possible reduction reactions involving Ti_3C_2 and $Ti_3C_2T_x$ MXene flakes

Since a reduction needs to take place during the lithiation of a lithium-ion negative electrode material it is important to identify the possible reduction reactions that may be seen when using $Ti_3C_2T_x$ MXene-based electrodes. Here it should be noted that the freestanding $Ti_3C_2T_x$ MXene electrodes used in this study only contained $Ti_3C_2T_x$ flakes since electrodes were made using a filtration of a suspension of $Ti_3C_2T_x$ MXene flakes.

Since a reduction reaction requires the presence of the oxidised form of a redox couple, one should first investigate which oxidised species can be expected to be present in these freestanding $Ti_3C_2T_x$ MXene electrodes. One can then start by considering the composition of a single $Ti_3C_2T_x$ MXene flake based on Figure 2 in the main text. Here it should be recalled, that the $Ti_3C_2T_x$ flakes were obtained from the corresponding MAX phase by etching away the aluminium. The so generated Ti_3C_2 flakes should then react with water according to the following redox reaction (where $T_x = (OH)_2$):

$Ti_3C_2 + 2\ H_2O = Ti_3C_2(OH)_2 + H_2$

As this reaction involves hydrogen evolution (due to the reduction of water), it is immediately clear that the Ti_3C_2 flakes must undergo an accompanying oxidation. The two half-cell reactions are then

$2\ H_2O + 2\ e^- = 2\ OH^- + H_2$ (reduction)

$Ti_3C_2 + 2\ OH^- = Ti_3C_2(OH)_2 + 2\ e^-$ (oxidation)

Since titanium is more easily oxidised than carbon (the relevant standard potentials are $Ti^{2+} + 2\ e^- = Ti$, $E^0 = 1.4$ V vs. Li^+/Li, and $CO_2 + 4\ H^+ + 4\ e^- = C + 2\ H_2O$, $E^0 = 3.2$ V vs. Li^+/Li, respectively) the abovementioned oxidation should mainly involve the titanium, and then predominantly the titanium present on the surfaces of the Ti_3C_2 flakes. As is illustrated in Figure 2 in the main text, the $Ti_3C_2T_x$ MXene flakes should therefore contain two types of titanium species. On the surfaces of the flakes there should be titanium species in which the titanium is bonding to both T_x and C, whereas the centre of the flakes should contain titanium only bonding to carbon. These two types of species will henceforth be referred to as T_x-Ti-C and Ti-C, respectively. As indicated above, the T_x-Ti-C species should include titanium in the form of Ti(II), Ti(III) and/or Ti(IV). As will be shown below, the oxidation state of the Ti-C titanium should, on the other hand, be zero. It should therefore be possible to reduce the titanium present in the T_x-Ti-C species, whereas the Ti-C titanium should be electrochemically inactive. The capacity should then depend on the oxidation state of the titanium in the T_x-Ti-C species. This should depend on how the $Ti_3C_2T_x$ MXene flakes were manufactured and stored.

According to thermodynamics the Ti_3C_2 flakes should react spontaneously with oxygen to eventually yield TiO_2 and carbon according to the reaction $Ti_3C_2 + 3\ O_2 = 3\ TiO_2 + 2\ C$. A corresponding reaction should also be seen for the T_x-Ti-C species present on the surfaces of the $Ti_3C_2T_x$ MXene flakes. To hinder this, the exposure of $Ti_3C_2T_x$ MXene flakes to oxygen is generally recommended to be kept at a minimum. Since the Ti_3C_2 flakes likewise should react spontaneously with water (to finally yield TiO_2 and carbon) according to the reaction $Ti_3C_2 + 6\ H_2O = 3\ TiO_2 + 2\ C + 6\ H_2$, the exposure of both Ti_3C_2 and $Ti_3C_2T_x$ to water should also be avoided.

The titanium in the $Ti_3C_2T_x$ MXene flakes may thus be present as Ti(0), Ti(II), Ti(III) or Ti(IV) depending on how far the (spontaneous) oxidation of the flakes has proceeded during the manufacturing and storage of the $Ti_3C_2T_x$ MXene material.

The sensitivity of the $Ti_3C_2T_x$ MXene flakes to oxidation by water and/or oxygen is most likely one important reason for the variations in the capacities seen for $Ti_3C_2T_x$ MXene based electrodes manufactured using different procedures.

In the presence of fluoride or chloride, the oxidation of the Ti_3C_2 formed taking place during the etching process may also involve the following reactions

$Ti_3C_2 + 2\ H^+ + 2\ Cl^- = Ti_3C_2(Cl)_2 + H_2$

$Ti_3C_2 + 2\ H^+ + 2\ F^- = Ti_3C_2(F)_2 + H_2$

Here it should be noted that the $Ti_3C_2(Cl)_2$ and $Ti_3C_2(F)_2$ would contain two Ti(0) and one Ti(II), in analogy with the $Ti_3C_2(OH)_2$ case mentioned above. In this case, two out of the three Ti in the $Ti_3C_2(Cl)_2$ or $Ti_3C_2(F)_2$ would hence remain electroinactive during the lithiation step. This indicates that the lithiation capacity of a $Ti_3C_2T_x$ MXene electrode should be relatively low as long as the titanium in the T_x-Ti-C species has not undergone further oxidation.

Reduction of Ti_3C_2, $Ti_3C_2F_2$ and $Ti_3C_2OH_2$
As is demonstrated in this section, the redox activity of the $Ti_3C_2T_x$ MXene flakes can be estimated using data for some model species.

As mentioned in the main text, density functional theory calculations (1) have indicated that a reduction of Ti_3C_2 to $Ti_3C_2Li_2$ should take place at about 0.62 V vs. Li^+/Li according to the following reaction:

$Ti_3C_2 + 2\ e^- + 2\ Li^+ = Ti_3C_2Li_2$.

The corresponding reduction potentials for $Ti_3C_2F_2$ and $Ti_3C_2OH_2$ (yielding $Ti_3C_2F_2Li_2$ and $Ti_3C_2OH_2Li_2$) were found to be 0.56 and 0.14 V vs. Li^+/Li, respectively, (1). These reduction potentials hence indicate that the lithiation capacities of $Ti_3C_2T_x$-based electrodes should stem from reductions taking place at potentials below 0.6 V vs Li^+/Li. In practice, even lower potentials would most likely be needed due to the presence of overpotentials. The latter could prevent the reaction from taking place within the potential window used in the present experiments. In this context it should be noted that the use of a too low cut-off limit can result in significant (unwanted) lithium (under)deposition.

In the $Ti_3C_2T_x$ reduction reactions discussed above it should be noted that it is the carbon in the $Ti_3C_2T_x$ that is reduced. This is further supported by the similar reduction potential obtained for the reduction of carbon to Li_2C_2, presented below. As titanium would be present as elemental titanium at potentials below about 1.2 V vs. Li^+/Li there should not be any reductions involving titanium below this potential.

Reduction of carbon
Carbon can be reduced to Li_2C_2 at about 0.3 V vs. Li^+/Li assuming the following reaction:

$2\ C + 2\ e^- + 2\ Li^+ = Li_2C_2$

The abovementioned standard potential was calculated using $\Delta G^0 = -nFE^0$ and the following ΔG^0 values: C (0 kJ mol^{-1}), Li^+ (-293 kJ mol^{-1}) (2) and Li_2C_2 (-66.5 kJ mol^{-1}) (3).

Since this E^0 value (i.e., 0.3 V vs. Li^+/Li) is similar to that of 0.62 V vs. Li^+/Li reported for Li_2C_2 (1), it is reasonable to assume that it is the carbon that is reduced also in the case of Ti_3C_2.

These data hence demonstrate that carbon is a poor oxidizing agent. This means that carbon should not be able to oxidize elemental titanium, and that the titanium present in Ti_3C_2 consequently should be in its elemental (i.e., Ti(0)) state. This also means that the Ti-C titanium present in the centre of the $Ti_3C_2T_x$ MXene flakes should be elemental. As a result, the Ti-C titanium should therefore not undergo any reduction.

2. Oxidation of $Ti_3C_2T_x$ and Ti_3C_2 by oxygen and/or water

As already indicated above, it has been shown (4) that $Ti_3C_2T_x$ exposed to oxygen undergoes an oxidation eventually yielding TiO_2 and free carbon. This can be schematically described using the following reaction:

$2\ Ti_3C_2(OH)_2 + 5\ O_2 = 6\ TiO_2 + 2\ H_2O + 4\ C$

In addition, it has been found (5) that $Ti_3C_2T_x$ also can be oxidised to TiO_2 by water. The oxidation with water can be schematically described using the following reactions:

$2\ Ti_3C_2O_2 + 11\ H_2O = 6\ TiO_2 + CO + CO_2 + 2\ CH_4 + 7\ H_2$

$2\ Ti_3C_2(OH)_2 + 11\ H_2O = 6\ TiO_2 + CO + CO_2 + 2\ CH_4 + 9\ H_2$

It should be noted that all these reactions are spontaneous (see below). As the oxidation of titanium to TiO_2 (i.e., Ti(IV)) involves the sequential loss of four electrons, the oxidation should involve at least Ti(II) and Ti(III) as intermediate species. This means that the titanium in the $Ti_3C_2T_x$ MXene flakes should be present as Ti(0) in the Ti-C layer, and as Ti(II), Ti(III) and/or Ti(IV), in the T_x-Ti-C surface layer, depending on the experimental conditions. In the $Ti_3C_2T_x$ MXene case, the T_x-Ti-C species present on the surface of the flakes should then protect the Ti-C in the centre of the $Ti_3C_2T_x$ flakes from undergoing oxidation.

As the T_x-Ti-C species present on the surfaces of the $Ti_3C_2T_x$ MXene flakes should contain Ti(II), Ti(III) and/or Ti(IV)), which should be able to undergo reduction reactions, the T_x-Ti-C species should contribute to the lithiation and delithiation capacities in the potential region investigated in this work. It is also reasonable to assume that the capacity due to the T_x-Ti-C species should depend on the employed manufacturing procedure and the time the electrode has been exposed to water and/or oxygen.

3. Reduction of the titanium present in the T_x-Ti-C surface layer

Reduction of different T_x-Ti-C species

The T_x-Ti-C surface species, formed as a result of an oxidation involving oxygen and/or water, may undergo reduction in a nonaqueous electrolyte containing lithium ions. While this will be illustrated below using TiO_2 as an example, many different oxidised titanium species may undergo reduction in the potential region investigated in this work. As these reactions most likely overlap, voltammograms without well-defined peaks and sloping chronopotentiograms should typically be seen.

In a nonaqueous electrolyte containing lithium ions, TiO_2 may undergo lithiation according to the following reaction:

$TiO_2 + x\ Li^+ + x\ e^- = Li_xTiO_2$

This reaction typically gives rise to a reduction and a corresponding oxidation at about 1.7 and 2.0 V vs. Li^+/Li, respectively (6).

In a nonaqueous electrolyte containing lithium ions, TiO_2 may be reduced to TiC if carbon is present, according to the following reaction:

$TiO_2 + 4\ e^- + 4\ Li^+ + C = TiC + 2\ Li_2O$

The standard potential for this reaction should be about 1.0 V vs. Li^+/Li. This E^0 value was obtained using $\Delta G^0 = -nFE^0$ and the following ΔG^0 values: TiO2 (-890 kJ mol^{-1}) (2), Li^+ (-293 kJ mol^{-1}) (2), C (0 kJ mol^{-1}), TiC (-181 kJ mol^{-1}) (2) and Li_2O (-561 kJ mol^{-1}) (2).

This reduction of TiO_2 to TiC could take place during the first lithiation step if the spontaneous oxidation of $Ti_3C_2T_x$ by oxygen or water has generated TiO_2 and carbon. In most cases the TiO_2 should, however, first undergo the abovementioned lithium insertion reaction yielding Li_xTiO_2, which then could be reduced to TiC in an analogous reduction reaction to that shown above.

In a nonaqueous electrolyte containing lithium ions, TiO_2 may also undergo a conversion reaction yielding Ti and Li_2O according to the following reaction:

$$TiO_2 + 4\ Li^+ + 4\ e^- = Ti + 2\ Li_2O$$

The standard potential for this reaction should be about 0.6 V vs. Li^+/Li. This E^0 value was obtained using $\Delta G^0 = -nFE^0$ and the following ΔG^0 values: TiO2 (-890 kJ mol^{-1}) (2), Li^+ (-293 kJ mol^{-1}) (2), Ti (0 kJ mol^{-1}), and Li_2O (-561 kJ mol^{-1}) (2).

In most cases the TiO_2 should, however, first undergo the abovementioned lithium insertion reaction yielding Li_xTiO_2. As the conversion reaction in this case would involve Li_xTiO_2 rather than TiO_2, the reduction potential would hence be different.

Reduction of Ti^{2+} to Ti(0)

The standard potential for the reaction, $Ti^{2+} + 2\ e^- = Ti$, is -1.8 V vs. Ag/AgCl (sat'd) [2], which corresponds to a potential of about 1.4 V vs. Li^+/Li. This means that titanium should undergo spontaneous oxidation in the presence of oxygen and/or water and that elemental titanium only should be present at potentials below about 1.4 V vs. Li^+/Li. It is therefore clear that the reduction of any oxidised Ti species (e.g., Ti(II), Ti(III) or Ti(IV)) should take place at potentials higher than about 1.4 V.

4. What is the oxidation state of the titanium in the $Ti_3C_2T_x$ MXene flakes?

As indicated above, the carbon in the Ti-C layer should be present in its elemental state. If the carbon is present in its elemental state, the titanium in the Ti-C layer must also be in the elemental state, for electroneutrality reasons. The titanium present in the T_x-Ti-C surface layer should, however, be present as Ti(II), Ti(III) or Ti(IV). This means that the average oxidation state of the titanium in $Ti_3C_2T_x$ MXene flake should be higher than zero and lower than four depending on how far the oxidation of the $Ti_3C_2T_x$ MXene has proceeded. As indicated above, the final oxidation products would be TiO_2 and carbon.

It is also reasonable to assume that the average titanium oxidation state may differ for different $Ti_3C_2T_x$ MXene flakes in the electrode depending on how exposed these flakes are to oxygen and/or water and the degree of restacking of the flakes. It is therefore reasonable to assume that a higher average titanium oxidation state should be seen with surface sensitive techniques compared to with more bulk sensitive techniques.

5. What is the capacity contribution due to double layer charging?

The contribution from double layer charging to the overall capacity can be estimated as follows. It is assumed that the $Ti_3C_2T_x$ material had a surface area of 20 $m^2\ g^{-1}$ (see the results presented in [4]), that the mass loading was 1.31 mg, and that the double layer capacitance was 10 µF cm^{-2}. The surface area of the electrode was then 20 $m^2\ g^{-1} \times 1.31 \times 10^{-3}$ g = $2.6 \cdot 10^{-2}$ m^2 = 262 cm^2. This gives a double layer capacitance of 10 µF $cm^{-2} \times 262\ cm^2$ = 2.6 mF which corresponds to a specific capacitance of 2.6×10^{-3} F / 1.31×10^{-3} g = 2.0 F g^{-1}. For a scan rate of 0.1 mV s^{-1} and a potential window of 3.3 V the scan time would be 3.3 V / (0.1×10^{-3} V s^{-1}) = 33000 s. The double

layer charging current would then be 2.6×10^{-3} F × 0.1×10^{-3} V s^{-1} = 2.6×10^{-7} A. As a result, the double layer charge would be 2.6×10^{-7} A × 33000 s = 8.6×10^{-3} C = 2.4×10^{-3} mAh, or about 2 mAh g^{-1}, assuming a rectangular shaped cyclic voltammogram. A comparison of this double layer charge of about 2 mAh g^{-1} with the capacities in Table S1 (see below) indicates that double layer charging could merely explain about 3 % of the lithiation capacity seen on the 15th cycle. The contribution is, in fact, most likely significantly smaller as it is unlikely that the total area of the particles calculated above can be accessed in the experiments. This indicates that the main part of the lithiation and delithiation capacities must have been due to redox reactions involving the T_x-Ti-C surface species present on the $Ti_3C_2T_x$ MXene flakes.

6. Estimation of the capacity associated with the formation of the SEI layer

During the lithiation step an SEI layer should be formed due to the reduction of the electrolyte, typically at potentials below about 1 V vs. Li^+/Li. As this SEI formation gives rise to an irreversible capacity it is interesting to try to estimate the magnitude of the irreversible capacity loss due to this effect.

This estimation was made based on the presence of an SEI layer with an assumed thickness of 20 nm, an assumed molar weight of 150 g mol^{-1} and an assumed density of 2 g cm^{-3}, as well as an electrode area of 0.785 x 20 = 15.7 cm^2 (obtained by assuming a surface roughness factor of 20). This gives a SEI layer with a volume of 3.1×10^{-5} cm^3 which combined with the density yield a SEI mass of about 6.2×10^{-5} g which in turn corresponds to about 4.1 x 10^{-7} mol. Using Faraday's law, assuming a two-electron process, a SEI formation charge of about 80 mC is then finally obtained.

When comparing the estimated SEI formation charge of about 80 mC with the total irreversible capacity loss of about 280 mC seen during the first three cycles (see Table S1 below) it becomes clear that the observed capacity loss, most likely, was too large to be explained by the SEI formation reaction alone. It is therefore reasonable to assume that the capacity loss seen on the first few cycles also included at least one additional reduction reaction.

In Table S1 below, the lithiation and delithiation capacities as well as the lost capacity and the accumulated lost capacities after each constant current cycle are shown for the first 15 cycles for a freestanding $Ti_3C_2T_x$ electrode dried at mild conditions (i.e., at 120 °C in vacuum for 16 h). After three cycles, the accumulated lost capacity was about 280 mC whereas the corresponding value after 15 cycles was about 344 mC. As indicated in the main text, the initial capacity losses were most likely due to a combination of SEI formation, water reduction as well as the inability to fully reform the oxidised titanium and carbon species on the delithiation (i.e., oxidation) step.

Table S1. The lithiation, delithiation and the accumulated lost capacities during the first 15 constant current cycles for a freestanding $Ti_3C_2T_x$ electrode dried at 120° C.

Cycle number	Lithiation capacity (mAh/g)	Delithiation capacity (mAh/g)	Lost capacity (mC)	Accumulated lost capacity (mC)
1	105.34	55.19	243.74	236
2	63.74	56.11	37.10	273
3	53.28	51.91	6.68	280
4	53.51	52.82	3.34	283
5	54.27	51.76	12.24	295
6	53.05	52.21	4.08	299
7	54.58	52.67	9.27	309

8	53.97	53.59	1.85	311
9	56.03	54.35	8.16	319
10	55.80	54.81	4.82	324
11	56.34	55.11	5.94	329
12	55.50	54.12	6.68	336
13	55.42	54.89	2.60	339
14	56.79	55.88	4.45	343
15	58.63	58.40	1.11	344

7. Irreversible capacity losses due to the presence of water

As discussed in the main text, it is reasonable to assume that water can be adsorbed on the (hydrophilic) surfaces of the $Ti_3C_2T_x$ flakes. The experimental results (see e.g., Table S1) in fact indicate that the irreversible capacity losses seen during the first three cycles involved reduction of water present in the freestanding $Ti_3C_2T_x$ electrodes. This hypothesis is supported by analogous results obtained with TiO_2 electrodes. (7) In the latter case it was found that H_2O/OH species adsorbed on TiO_2 surfaces gave rise to an irreversible capacity via the generation of Li_2O. Given the fact that the surfaces of present freestanding $Ti_3C_2T_x$ electrodes should contain oxidised titanium species after being exposed to water and/or air, a similar irreversible capacity effect should also be seen here.

While the estimation of the capacity loss due to SEI formation above indicated that this loss should be of the order of 80 mC, the total capacity loss during the first three cycles was 280 mC. It can then be assumed that the difference between these values (i.e., 280 - 80 = 200 mC) was due to the reduction of the adsorbed water according to the following reaction:

$2\ H_2O + 4\ e^- + 4\ Li^+ = 2\ H_2 + 2\ Li_2O$

A charge of 200 mC would correspond to about 1.0 x 10^{-6} mol of water, i.e., about 19 μg of water. For an electrode mass loading of 1.31 mg, this would correspond to a concentration of water in the $Ti_3C_2T_x$ electrode of about 1.4 wt.%.

As mentioned above it is, however, also possible that there was a capacity loss due to the inability to fully regenerate the oxidised titanium and carbon species on the delithiation step. If so, the water concentration in the $Ti_3C_2T_x$ electrode should clearly have been lower than about 1.4 wt.%.

8. SEM cross-section image

The cross-section SEM image in Figure S2, showing the morphology of the pristine electrode and individual flakes, was taken on a Zeiss Merlin SEM with an acceleration voltage of 5.00 kV, a current of 100 pA, a working distance of 4.1 mm and with the in-lens detector.

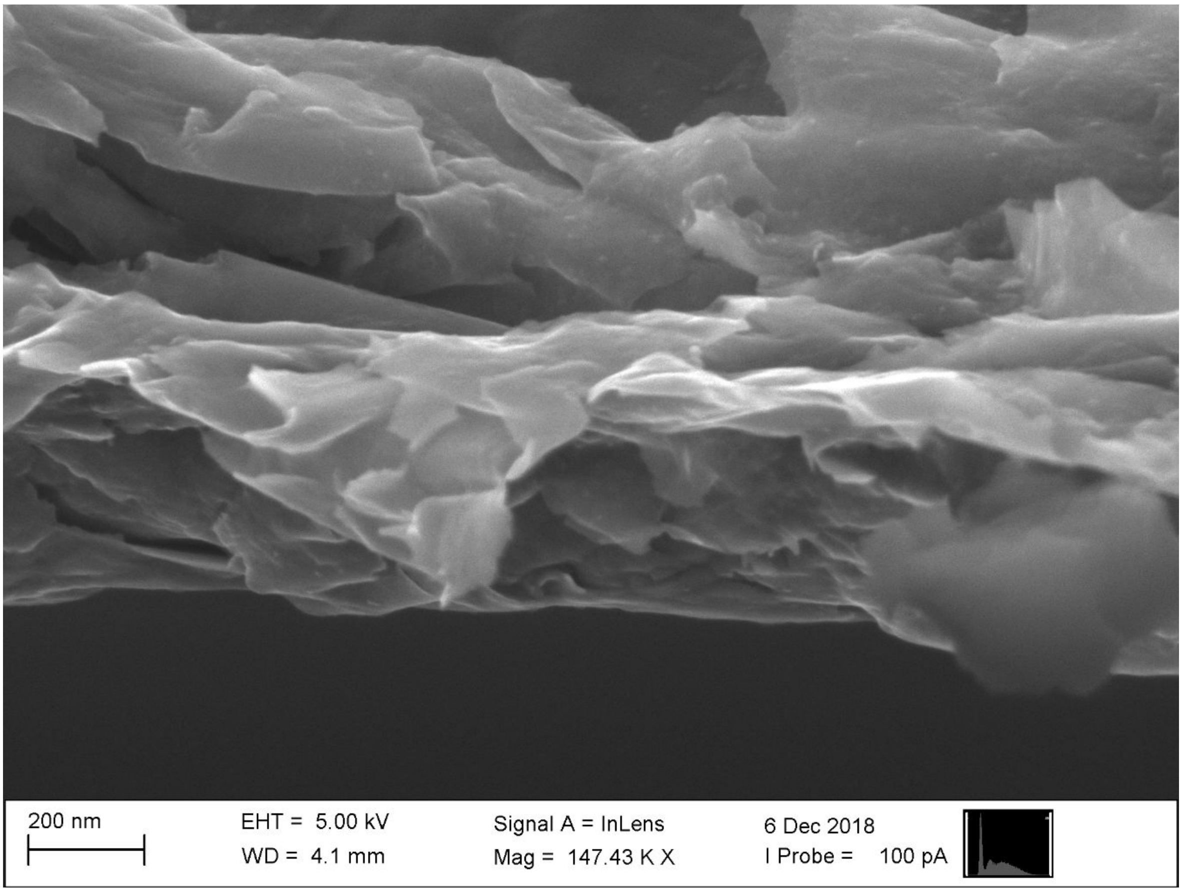

Figure S1. Cross-section SEM of a pristine multi-layered delaminated freestanding $Ti_3C_2T_x$ electrode.

9. Cycling curves for a $Ti_3C_2T_x$ electrode dried at 120 °C

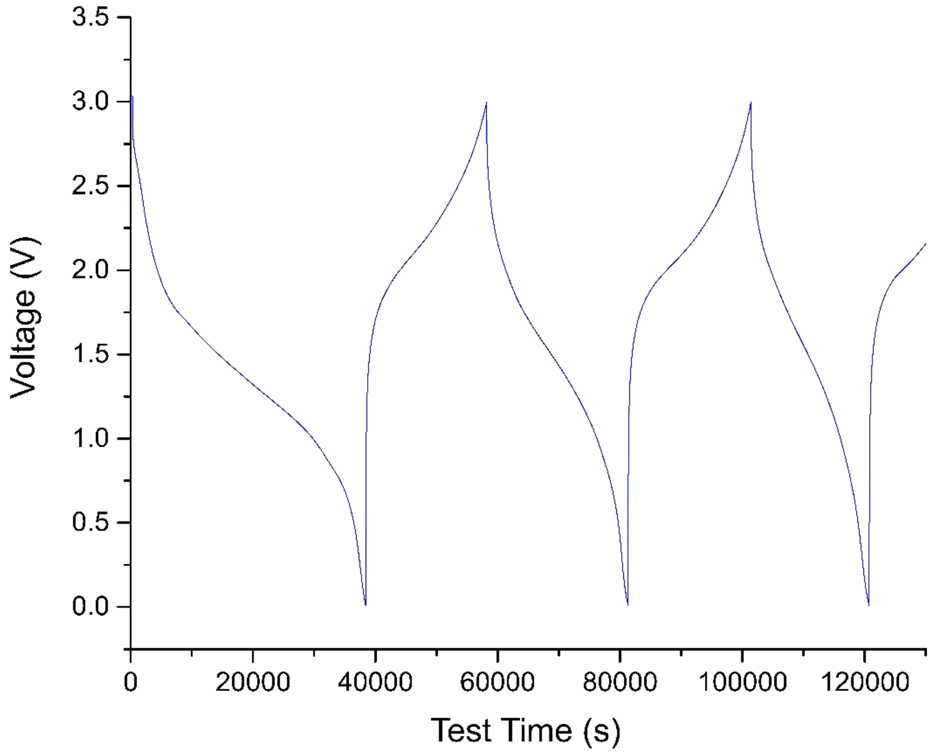

Figure S2. Constant current cycling curves for a freestanding $Ti_3C_2T_x$ electrode, dried at 120 °C for 16 hours, recorded using a current density of 10 mA g^{-1}.

10. Voltammetric lithiation and delithiation capacities for a $Ti_3C_2T_x$ electrode dried at 120 °C

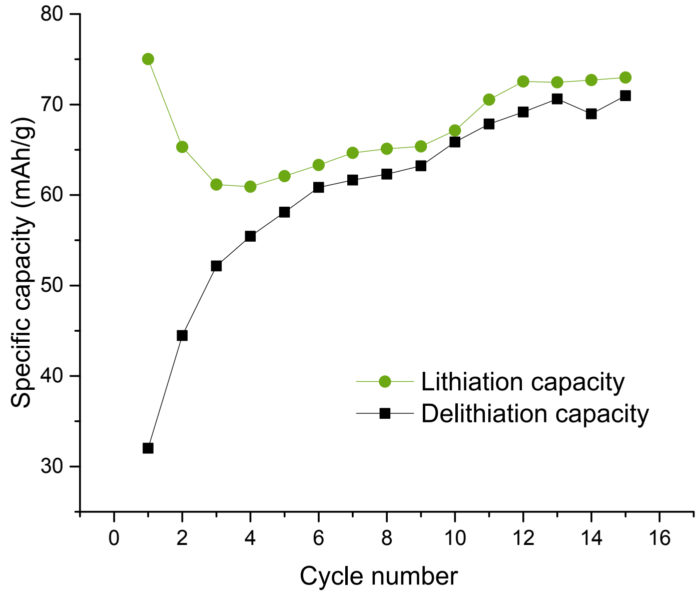

Figure S3. The lithiation (i.e., reduction) and delithiation (i.e., oxidation) capacities as a function of the cycle number for a freestanding $Ti_3C_2T_x$ electrode using cycling voltammetry at a scan rate of 0.1 mV s^{-1}. The electrode mass loading was 1.35 mg.

12. HAXPES spectra for one rested and two cycled electrodes

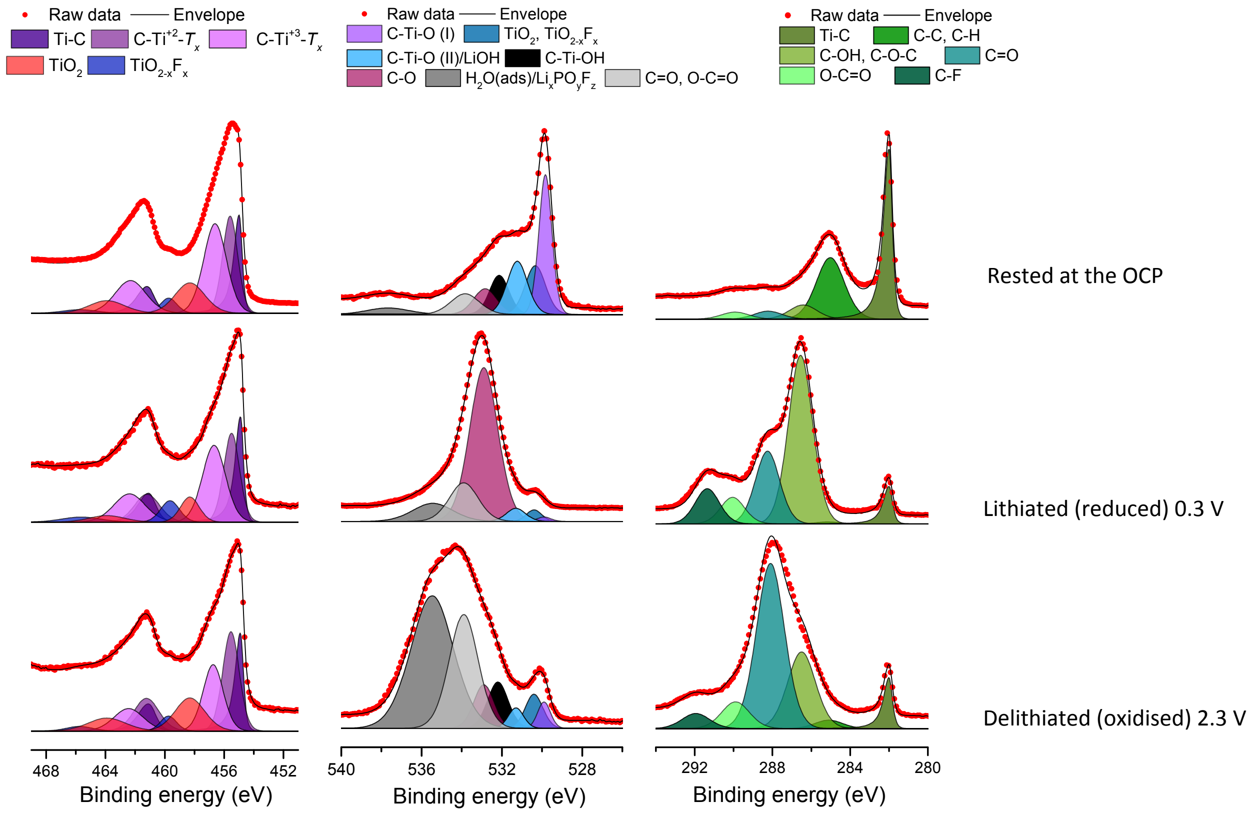

Figure S4. HAXPES spectra of Ti2p, O1s and C1s edges for a rested at the OCP, a lithiated (i.e., reduced) and a delithiated (i.e., oxidised) $Ti_3C_2T_x$ electrode, respectively. The spectra were recorded at the indicated potentials on the first cycle.

13. Ti K-edge energies

Table S2. Ti K-edge energies obtained from transmission XAS experiments

	Ti K-edge, eV
**Ti foil**	4966.4
**Pristine $Ti_3C_2T_x$**	4978.9
**Rested at OCP $Ti_3C_2T_x$**	4979.9
**Lithiated to 0.3 V 1st cycle**	4979.4
**Lithiated to 2.75 V 81st cycle**	4979.9
**Oxidised in water $Ti_3C_2T_x$**	4980.4
**TiO_2 (anatase)**	4984.8

The energy of the Ti K-edge was measured at half the height of the normalized edge.

14. XPS spectra for oxidised film electrodes

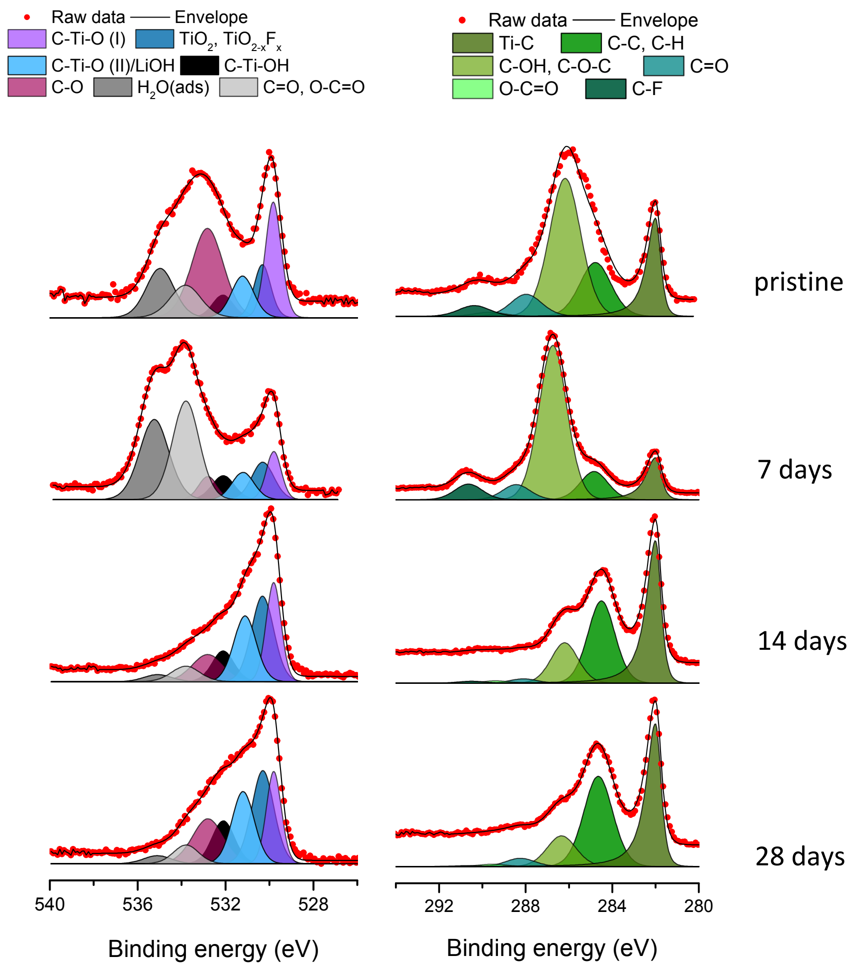

Figure S5. XPS spectra for the O1s and C1s edges for a pristine freestanding $Ti_3C_2T_x$ film as well as freestanding films prepared using a vial with $Ti_3C_2T_x$ suspension exposed to air for 7, 14 and 28 days, respectively.

15. Cycling curves for a $Ti_3C_2T_x$ electrode dried at 300 °C

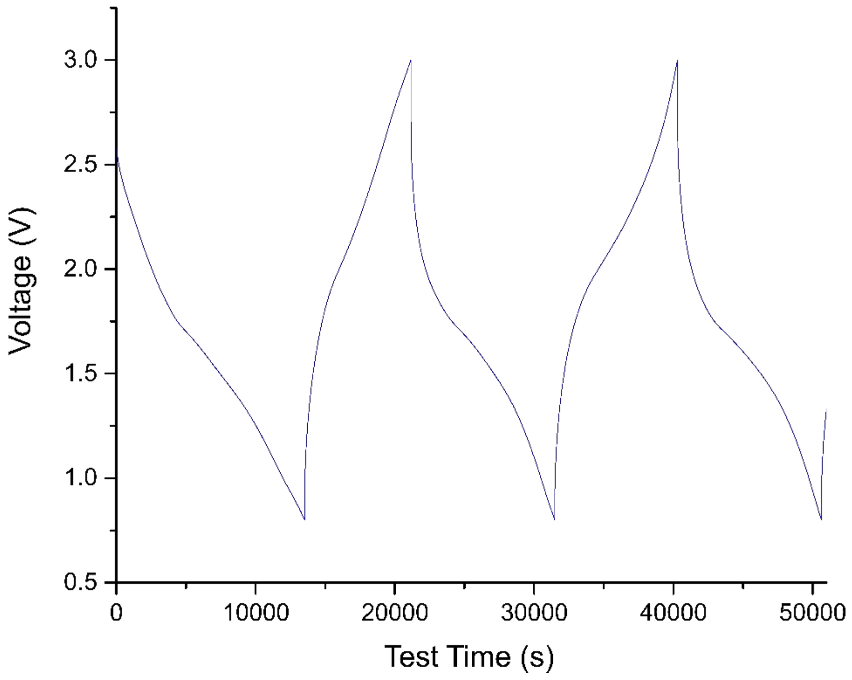

Figure S6. Constant current cycling curves for a freestanding $Ti_3C_2T_x$ electrode, dried at 300 °C for 16 hours, recorded using a current density of 10 mA g^{-1}.

16. Ti $L_{2,3}$- edge XAS spectra for cycled and rested at OCP electrodes

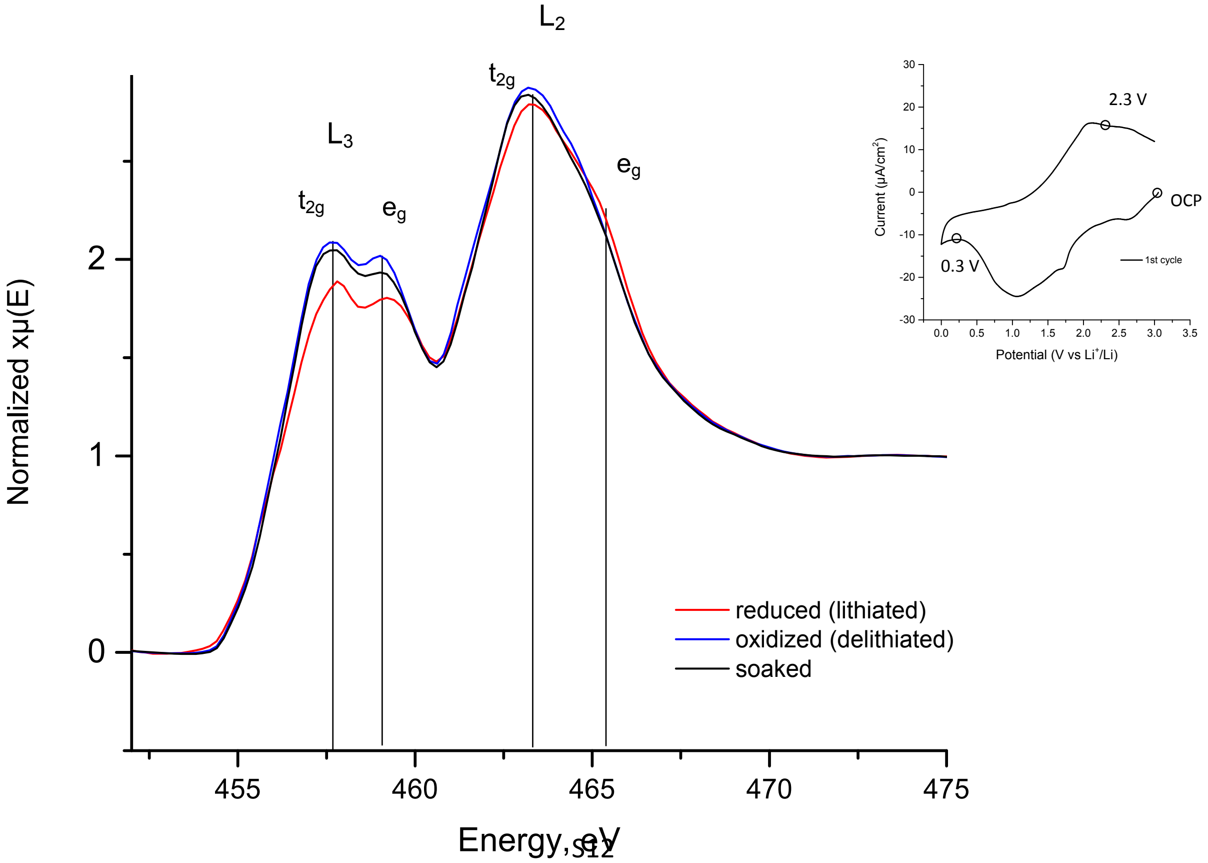

Figure S7. Ti $L_{2,3}$- edge XAS spectra for one soaked (rested at OCP) and two freestanding $Ti_3C_2T_x$ electrodes cycled against a lithium electrode in an electrolyte composed of 1 M $LiPF_6$ in 1:1 EC:DEC. The inset shows the cyclic voltammogram and the points at which the cycling was stopped prior to the XAS measurements. The lithiated (i.e., reduced) and delithiated (i.e., oxidised) electrodes were hence stopped at 0.3 and 2.3 V vs. Li^+/Li, respectively. The rested at OCP electrode exhibited an open circuit potential of about 3.2 V vs. Li^+/Li.

The L-edge XAS spectra for the rested at OCP and cycled $Ti_3C_2T_x$ electrodes in Figure S11 feature two bands: L_3 and L_2, in analogy with the spectra for bulk TiC (8). For the delithiated electrode oxidized to 2.3 V vs. Li^+/Li and the rested at OCP $Ti_3C_2T_x$ electrode, the L_3 band could be resolved into two peaks, corresponding to the t_{2g} and e_g bands, located at 457.6 and 459.0 eV. For the lithiated electrode cycled to 0.3 V vs. Li^+/Li, the bands were slightly shifted toward higher energies, i.e., 457.8 and 459.2 eV, respectively. This was unexpected as the energy of the Ti L-edge tends to shift to higher energies for higher Ti valence states. The effect may, however, have been caused by a change in the termination from -OH and =O to -F upon the lithiation(reduction).

The L_2 band for all three electrodes features a maximum and a faint shoulder (corresponding to the t_{2g} and e_g bands) situated at 463.2 and 464.4 eV for the delithiated electrode cycled to 2.3 V vs. Li^+/Li, at 463.1 and 464.4 eV for the rested at OCP non-cycled electrode and at 463.2 and 465.1 eV for the (lithiated) electrode cycled to 0.3 V vs. Li^+/Li.

The e_g band should be sensitive to the local environment as the Ti e_g orbitals are believed to point directly to the 2p orbitals of the surrounding O atoms in a MXene.

For the L_3 band, the ratio between the e_g and t_{2g} peak intensities was the same, i.e., 0.93, for the rested at OCP electrode and the lithiated electrode cycled to 0.3 V vs. Li^+/Li. For the delithiated electrode cycled to 2.3 V vs. Li^+/Li the ratio was somewhat higher, i.e., 0.95. For the L_2 band, the ratio between the e_g and t_{2g} peak intensities was 0.87 for the rested at OCP electrode, 0.8 for the lithiated electrode and 0.88 for the delithiated electrode. This small change in the e_g/t_{2g} ratio suggests a small change in the titanium oxidation state at the surface (i.e., within a depth of a few nanometres) of the electrodes during the first cycle.

17. XPS peak ratios for different electrodes

Table S3. Ratios between the main XPS peaks' areas extracted from fits made with the CasaXPS software (9).

ratio between areas of the peaks [region]/ sample id	Ti(+2) [Ti 2p] / Ti-C [Ti 2p]	Ti(+3) [Ti 2p] / Ti-C [Ti 2p]	(TiO_2 + $TiO_{2-x}F_x$) [Ti 2p] /Ti-C [Ti 2p]	Ti-C [C 1s] / Ti-C [Ti 2p]	C-Ti-O(I) [O 1s] /Ti-C [Ti 2p]	C-Ti-OH [O 1s] /Ti-C [Ti 2p]
pristine	2.43	3.26	1.65	1.28	0.75	0.19
rested at OCP	1.62	3.15	1.32	1.07	0.67	0.32
lit (red) 0.3 V	2.03	2.44	0.68	1.29	0.00	0.00
delith (ox) 2.3 V	2.55	3.80	1.62	1.60	0.76	1.45
HAXPES rested at OCP	1.43	1.98	1.13	0.42	0.41	0.17
HAXPES lit (red) 0.3 V	1.28	1.62	0.79	0.36	0.19	0.00
HAXPES delith (ox) 2.3 V	1.61	1.30	1.19	0.43	0.28	0.77
HT300 pristine	2.35	4.24	1.97	1.58	1.20	19.76
HT300 rested at OCP	1.36	2.98	1.83	1.14	0.73	2.30

HT300 lit (red) 0.3 V	1.96	2.35	0.94	1.22	0.00	0.01
HT300 delith (ox) 2.3 V	3.18	4.50	1.77	1.89	0.86	2.66
7 days ox	1.82	3.07	1.51	1.26	0.52	0.38
14 days ox	1.90	3.33	2.05	1.23	0.63	0.29
28 days ox	1.76	3.24	1.90	1.20	0.57	0.40

18. XPS fitting details

Table S4. XPS peak fitting results for Ti 2p region of various $Ti_3C_2T_x$ MXenes electrodes.

Sample	BE, eV (2p 3/2)	BE, eV (2p 1/2)	FWHM, eV (2p 3/2)	FWHM, eV (2p 1/2)	Fraction	Assigned to
pristine	454.9	461.1	0.6	1.1	0.12	Ti-C
	455.5	461.2	1.3	2.0	0.29	C-Ti(+2)-T_x
	456.6	462.3	1.9	2.4	0.39	C-Ti(+3)-T_x
	458.3	463.9	2.4	2.7	0.17	TiO_2
	459.6	465.6	1.5	3.2	0.03	TiO_{2-xFx}
rested at OCP	455.0	461.2	0.6	1.1	0.14	Ti-C
	455.5	461.2	1.2	2.0	0.23	C-Ti(+2)-T_x
	456.6	462.3	2.0	2.4	0.44	C-Ti(+3)-T_x
	458.3	463.9	1.9	2.0	0.13	TiO_2
	459.8	465.8	1.6	2.8	0.06	TiO_{2-xFx}
lit (red) 0.3 V	454.8	461.0	0.6	1.3	0.16	Ti-C
	455.3	461.0	1.3	2.0	0.33	C-Ti(+2)-T_x
	456.6	462.3	2.0	2.5	0.40	C-Ti(+3)-T_x
	458.3	463.9	1.3	3.0	0.05	TiO_2
	459.2	465.2	1.7	3.2	0.07	TiO_{2-xFx}
delith (ox) 2.3 V	454.9	461.1	0.5	1.0	0.16	Ti-C
	455.4	461.1	1.3	2.0	0.33	C-Ti(+2)-T_x
	456.5	462.2	1.9	2.5	0.40	C-Ti(+3)-T_x
	458.3	463.9	2.0	2.8	0.05	TiO_2
	459.9	465.9	1.5	3.2	0.07	TiO_{2-xFx}
HAXPES rested at OCP	455.0	461.2	0.5	1.2	0.18	Ti-C
	455.6	461.3	1.2	2.0	0.26	C-Ti(+2)-T_x
	456.6	462.3	1.8	2.5	0.36	C-Ti(+3)-T_x
	458.3	463.9	2.4	2.8	0.16	TiO_2
	459.7	465.7	1.3	2.7	0.04	TiO_{2-xFx}
HAXPES lit (red) 0.3 V	454.9	461.1	0.5	1.2	0.21	Ti-C
	455.5	461.2	1.3	2.0	0.27	C-Ti(+2)-T_x
	456.7	462.4	1.8	2.5	0.34	C-Ti(+3)-T_x
	458.3	463.9	1.5	2.8	0.09	TiO_2
	459.6	465.6	1.5	3.1	0.08	TiO_{2-xFx}
HAXPES delith (ox) 2.3 V	454.9	461.1	0.5	1.2	0.20	Ti-C
	455.5	461.2	1.3	2.0	0.31	C-Ti(+2)-T_x
	456.7	462.4	1.6	2.4	0.26	C-Ti(+3)-T_x
	458.3	463.9	2.4	3.0	0.19	TiO_2
	459.8	465.8	1.3	2.1	0.05	TiO_{2-xFx}
HT300 pristine	455.0	461.2	0.5	1.0	0.10	Ti-C
	455.5	461.2	1.2	1.9	0.25	C-Ti(+2)-T_x
	456.6	462.3	2.0	2.5	0.44	C-Ti(+3)-T_x
	458.3	463.9	2.1	3.0	0.15	TiO_2
	459.7	465.7	1.5	3.2	0.06	TiO_{2-xFx}
HT300 rested at OCP	455.0	461.2	0.6	1.1	0.14	Ti-C
	455.6	461.3	1.2	2.0	0.19	C-Ti(+2)-T_x
	456.5	462.2	2.0	2.4	0.42	C-Ti(+3)-T_x
	458.3	463.9	2.5	2.5	0.17	TiO_2
	459.8	465.8	1.4	2.4	0.08	TiO_{2-xFx}

Sample	BE, eV (2p 3/2)	BE, eV (2p 1/2)	FWHM, eV (2p 3/2)	FWHM, eV (2p 1/2)	Fraction	Assigned to
HT300 lit (red) 0.3 V	454.9	461.1	0.6	1.3	0.16	Ti-C
	455.4	461.1	1.3	2.0	0.31	C-Ti(+2)-T_x
	456.7	462.4	2.0	2.4	0.38	C-Ti(+3)-T_x
	458.5	464.1	1.6	3.0	0.11	TiO_2
	459.9	465.9	1.5	2.8	0.04	TiO_{2-xFx}
HT300 delith (ox) 2.3 V	455.0	461.2	0.5	1.1	0.10	Ti-C
	455.5	461.2	1.3	1.9	0.30	C-Ti(+2)-T_x
	456.6	462.3	1.9	2.5	0.43	C-Ti(+3)-T_x
	458.3	463.9	1.8	3.0	0.13	TiO_2
	459.6	465.6	1.2	3.2	0.04	TiO_{2-xFx}
7 days ox	455.0	461.2	0.6	1.1	0.14	Ti-C
	455.5	461.2	1.3	2.0	0.25	C-Ti(+2)-T_x
	456.6	462.3	2.0	2.4	0.41	C-Ti(+3)-T_x
	458.3	463.9	2.1	2.3	0.13	TiO_2
	459.7	465.7	1.5	2.7	0.07	TiO_{2-xFx}
14 days ox	455.0	461.2	0.6	1.1	0.12	Ti-C
	455.5	461.2	1.3	2.0	0.23	C-Ti(+2)-T_x
	456.6	462.3	2.0	2.4	0.40	C-Ti(+3)-T_x
	458.3	463.9	2.4	2.8	0.18	TiO_2
	459.7	465.7	1.3	2.6	0.07	TiO_{2-xFx}
28 days ox	455.0	461.2	0.6	1.1	0.13	Ti-C
	455.6	461.3	1.3	2.0	0.22	C-Ti(+2)-T_x
	456.6	462.3	2.0	2.4	0.41	C-Ti(+3)-T_x
	458.3	463.9	2.1	2.4	0.15	TiO_2
	459.7	465.7	1.4	2.4	0.09	TiO_{2-xFx}

Table S5. XPS peak fitting results for O 1s region of various $Ti_3C_2T_x$ MXenes electrodes.

Sample	BE, eV	FWHM, eV	Fraction	Assigned to
pristine	529.8	0.8	0.18	C-Ti-O (I)
	530.3	0.9	0.09	TiO_2, $TiO_{2-x}F_x$
	531.2	1.2	0.09	C-Ti-O (II)/LiOH
	532.1	1.1	0.04	C-Ti-OH
	532.8	1.7	0.27	C-OH, C-O-C, C-O
	533.8	1.7	0.19	C=O, O-C=O
	535.0	1.5	0.13	H_2O(ads)
rested at OCP	529.8	0.8	0.15	C-Ti-O (I)
	530.3	0.9	0.07	TiO_2, $TiO_{2-x}F_x$
	531.2	1.2	0.11	C-Ti-O (II)/LiOH
	532.1	1.0	0.07	C-Ti-OH
	532.8	1.4	0.24	C-OH, C-O-C, C-O
	533.8	1.7	0.30	C=O, O-C=O
	535.0	1.4	0.06	H_2O(ads)
lit (red) 0.3 V	529.5	0.8	0.00	C-Ti-O (I)
	530.0	0.9	0.01	TiO_2, $TiO_{2-x}F_x$
	530.9	1.2	0.02	C-Ti-O (II)/LiOH
	531.8	1.1	0.00	C-Ti-OH
	532.5	1.4	0.07	C-OH, C-O-C, C-O
	533.5	1.3	0.38	C=O, O-C=O
	534.7	2.2	0.52	H_2O(ads)/$Li_xPO_yF_z$
delith (ox) 2.3 V	529.8	0.9	0.07	C-Ti-O (I)
	530.3	0.9	0.06	TiO_2, $TiO_{2-x}F_x$
	531.2	1.2	0.05	C-Ti-O (II)/LiOH
	532.1	1.2	0.13	C-Ti-OH
	532.8	1.7	0.40	C-OH, C-O-C, C-O
	533.8	1.7	0.17	C=O, O-C=O
	534.8	1.9	0.12	H_2O(ads)/$Li_xPO_yF_z$
HAXPES rested at OCP	529.8	0.8	0.30	C-Ti-O (I)
	530.3	1.2	0.16	TiO_2, $TiO_{2-x}F_x$
	531.2	1.2	0.18	C-Ti-O (II)/LiOH
	532.1	1.1	0.12	C-Ti-OH
	532.8	1.4	0.10	C-OH, C-O-C, C-O
	533.8	1.7	0.10	C=O, O-C=O
	537.7	2.5	0.05	H_2O(ads)
HAXPES lit (red) 0.3 V	529.9	0.8	0.01	C-Ti-O (I)
	530.4	0.9	0.03	TiO_2, $TiO_{2-x}F_x$
	531.3	1.1	0.04	C-Ti-O (II)/LiOH
	532.2	1.1	0.00	C-Ti-OH
	532.9	1.7	0.65	C-OH, C-O-C, C-O
	533.9	1.7	0.17	C=O, O-C=O
	535.4	2.4	0.11	H_2O(ads)/$Li_xPO_yF_z$
HAXPES delith (ox) 2.3 V	529.9	0.8	0.03	C-Ti-O (I)
	530.4	1.0	0.05	TiO_2, $TiO_{2-x}F_x$
	531.3	0.9	0.03	C-Ti-O (II)/LiOH
	532.2	1.2	0.08	C-Ti-OH

	532.9	1.2	0.07	C-OH, C-O-C, C-O
	533.9	1.7	0.27	C=O, O-C=O
Sample	BE, eV	FWHM, eV	Fraction	Assigned to
HAXPES delith (ox) 2.3 V	535.5	2.5	0.47	H_2O(ads)/$Li_xPO_yF_z$
HT300 pristine	529.9	0.8	0.03	C-Ti-O (I)
	530.4	0.9	0.01	TiO_2, $TiO_{2-x}F_x$
	531.3	1.0	0.07	C-Ti-O (II)/LiOH
	532.2	1.2	0.46	C-Ti-OH
	532.9	0.8	0.05	C-OH, C-O-C, C-O
	533.9	1.7	0.37	C=O, O-C=O
HT300 rested at OCP	529.9	0.8	0.05	C-Ti-O (I)
	530.4	1.2	0.04	TiO_2, $TiO_{2-x}F_x$
	531.3	1.2	0.06	C-Ti-O (II)/LiOH
	532.2	1.1	0.15	C-Ti-OH
	532.9	0.9	0.08	C-OH, C-O-C, C-O
	533.9	1.6	0.38	C=O, O-C=O
	535.5	1.6	0.24	H_2O(ads)
HT300 lit (red) 0.3 V	529.5	0.8	0.00	C-Ti-O (I)
	530.0	0.9	0.02	TiO_2, $TiO_{2-x}F_x$
	530.9	1.2	0.02	C-Ti-O (II)/LiOH
	531.8	1.1	0.00	C-Ti-OH
	532.5	1.5	0.07	C-OH, C-O-C, C-O
	533.5	1.5	0.43	C=O, O-C=O
	534.9	1.8	0.47	H_2O(ads)/$Li_xPO_yF_z$
HT300 delith (ox) 2.3 V	529.9	0.9	0.04	C-Ti-O (I)
	530.4	1.0	0.04	TiO_2, $TiO_{2-x}F_x$
	531.3	0.9	0.03	C-Ti-O (II)/LiOH
	532.2	1.1	0.12	C-Ti-OH
	532.9	1.3	0.28	C-OH, C-O-C, C-O
	533.9	1.7	0.21	C=O, O-C=O
	534.3	1.9	0.28	H_2O(ads)/$Li_xPO_yF_z$
7 days ox	529.8	0.8	0.09	C-Ti-O (I)
	530.3	1.2	0.10	TiO_2, $TiO_{2-x}F_x$
	531.2	1.2	0.08	C-Ti-O (II)/LiOH
	532.1	1.2	0.07	C-Ti-OH
	532.8	1.0	0.05	C-OH, C-O-C, C-O
	533.8	1.4	0.33	C=O, O-C=O
	535.2	1.6	0.29	H_2O(ads)
14 days ox	529.8	0.8	0.21	C-Ti-O (I)
	530.3	1.2	0.27	TiO_2, $TiO_{2-x}F_x$
	531.1	1.2	0.21	C-Ti-O (II)/LiOH
	532.1	1.2	0.10	C-Ti-OH
	532.8	1.7	0.12	C-OH, C-O-C, C-O
	533.8	1.5	0.06	C=O, O-C=O
	535.1	1.5	0.03	H_2O(ads)
28 days ox	529.8	0.8	0.17	C-Ti-O (I)
	530.3	1.2	0.26	TiO_2, $TiO_{2-x}F_x$
	531.2	1.2	0.20	C-Ti-O (II)/LiOH
	532.1	1.2	0.12	C-Ti-OH

	532.8	1.7	0.17	C-OH, C-O-C, C-O
	533.8	1.4	0.06	C=O, O-C=O
	535.1	1.5	0.03	H_2O(ads)

Table S6. XPS peak fitting results for C 1s region of various Ti3C2*Tx* MXenes electrodes.

Sample	BE, eV	FWHM, eV	Fraction	Assigned to
pristine	282.0	0.6	0.19	Ti-C
	284.8	1.7	0.19	C-C, C-H
	286.2	1.7	0.49	C-OH, C-O-C
	288.0	1.7	0.08	C=O
	289.7	1.7	0.01	O-C=O
	290.4	1.7	0.04	C-F
rested at OCP	282.0	0.6	0.15	Ti-C
	285.3	1.6	0.53	C-C, C-H
	286.5	1.6	0.25	C-OH, C-O-C
	288.2	1.6	0.03	C=O
	289.6	1.6	0.04	O-C=O
	291.0	1.6	0.00	C-F
lit (red) 0.3 V	282.0	0.7	0.02	Ti-C
	284.7	1.5	0.00	C-C, C-H
	286.4	1.5	0.79	C-OH, C-O-C
	288.1	1.5	0.09	C=O
	289.2	1.5	0.02	O-C=O
	290.5	1.5	0.08	C-F
delith (ox) 2.3 V	282.0	0.6	0.16	Ti-C
	284.6	1.4	0.02	C-C, C-H
	285.8	1.4	0.63	C-OH, C-O-C
	287.6	1.4	0.11	C=O
	289.5	1.4	0.05	O-C=O
	290.6	1.4	0.03	C-F
HAXPES rested at OCP	282.0	0.4	0.42	Ti-C
	285.0	1.7	0.39	C-C, C-H
	286.4	1.7	0.09	C-OH, C-O-C
	288.2	1.7	0.05	C=O
	289.9	1.7	0.05	O-C=O
	290.6	1.7	0.00	C-F
HAXPES lit (red) 0.3 V	282.0	0.4	0.06	Ti-C
	285.2	1.5	0.01	C-C, C-H
	286.6	1.5	0.52	C-OH, C-O-C
	288.3	1.5	0.23	C=O
	290.1	1.5	0.08	O-C=O
	291.4	1.5	0.11	C-F
HAXPES delith (ox) 2.3 V	282.0	0.4	0.06	Ti-C
	285.1	1.7	0.03	C-C, C-H
	286.5	1.7	0.25	C-OH, C-O-C
	288.1	1.7	0.53	C=O
	289.9	1.7	0.09	O-C=O
	291.9	1.7	0.05	C-F
HT300 pristine	282.0	0.6	0.24	Ti-C

	284.7	1.4	0.16	C-C, C-H
	285.7	1.4	0.43	C-OH, C-O-C
	287.3	1.4	0.18	C=O
HT300 rested at OCP	282.0	0.6	0.05	Ti-C
	284.9	1.3	0.12	C-C, C-H
	285.6	1.3	0.09	C-OH, C-O-C
Sample	BE, eV	FWHM, eV	Fraction	Assigned to
HT300 rested at OCP	287.0	1.3	0.60	C=O
	288.6	1.3	0.07	O-C=O
	290.9	1.3	0.06	C-F
HT300 lit (red) 0.3 V	282.0	0.6	0.02	Ti-C
	284.6	1.4	0.01	C-C, C-H
	286.3	1.4	0.79	C-OH, C-O-C
	288.0	1.4	0.11	C=O
	289.0	1.4	0.00	O-C=O
	290.2	1.4	0.07	C-F
HT300 delith (ox) 2.3 V	282.0	0.6	0.06	Ti-C
	284.8	1.2	0.03	C-C, C-H
	285.3	1.2	0.77	C-OH, C-O-C
	287.0	1.2	0.09	C=O
	288.0	1.2	0.04	O-C=O
	290.0	1.2	0.02	C-F
7 days ox	282.0	0.6	0.12	Ti-C
	284.8	1.5	0.12	C-C, C-H
	286.7	1.5	0.64	C-OH, C-O-C
	288.4	1.5	0.06	C=O
	289.8	1.5	0.00	O-C=O
	290.6	1.5	0.06	C-F
14 days ox	282.0	0.6	0.42	Ti-C
	284.5	1.5	0.36	C-C, C-H
	286.2	1.5	0.18	C-OH, C-O-C
	288.1	1.5	0.02	C=O
	289.4	1.5	0.01	O-C=O
	290.5	1.5	0.01	C-F
28 days ox	282.0	0.6	0.40	Ti-C
	284.6	1.6	0.41	C-C, C-H
	286.3	1.6	0.14	C-OH, C-O-C
	288.2	1.6	0.04	C=O
	289.5	1.6	0.01	O-C=O
	290.6	1.6	0.00	C-F

19. EIS results

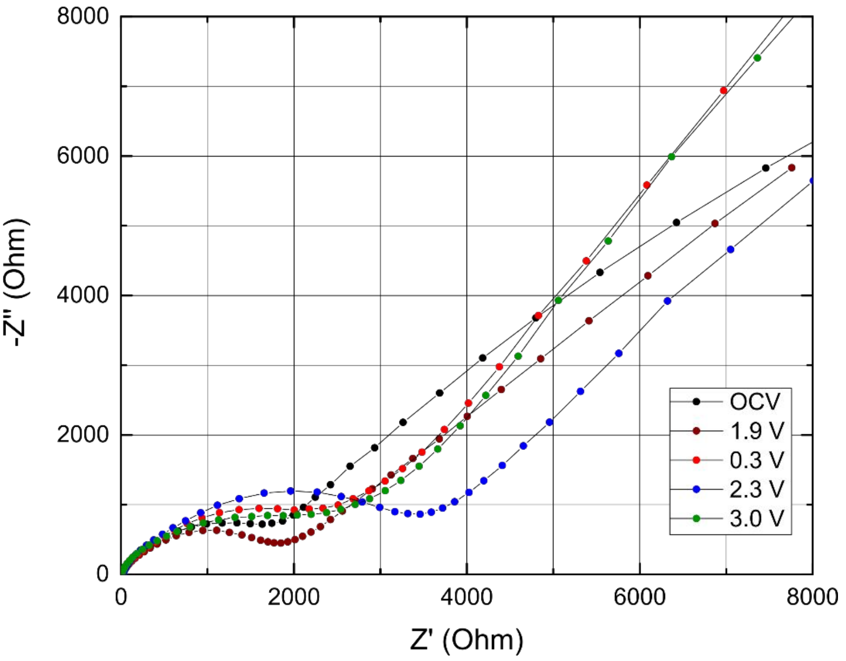

Figure S8. EIS spectra (dots) recorded between 20 kHz and 5 mHz at different potentials, i.e., the OCV (~3 V), 1.9 and 0.3 V vs. Li^+/Li on lithiation (i.e., reduction) as well as 2.3 and 3 V vs. Li^+/Li on delithiation (i.e., oxidation) using a $Ti_3C_2T_x$/Li cell. The ac amplitude was 10 mV.

In Figure S8, the ac impedance was first measured at the OCV (~3 V vs. Li^+/Li) and then after scanning the potential (at a scan rate 0.1 mV s^{-1}) to 1.9 V vs. Li^+/Li and 0.3 V vs. Li^+/Li, and subsequently to 2.3 V vs. Li^+/Li and 3.0 V vs. Li^+/Li. The cell was held at each of the abovementioned potentials for 30 minutes prior to the ac measurement.

The shapes of the Nyquist plots were clearly dependent on the dc potential (i.e., the state of charge) of the electrode indicating the presence of potential dependent redox reactions. This is in good agreement with the cyclic voltammetry and chronopotentiometric results presented in the main text.

References:

(1) Tang, Q.; Zhou, Z.; Shen, P. Are MXenes promising anode materials for Li ion batteries? Computational studies on electronic properties and Li storage capability of Ti_3C_2 and $Ti_3C_2X_2$ (X = F, OH) monolayer. *J. Am. Chem. Soc.* **2012,** *134*, 16909–16916.

(2) Aylward G. H.; Findlay T. J. V. SI Chemical Data, 5th ed.; John Wiley and Sons: Milton, Queensland, 2002; pp 1-202.

(3) Sharafat, S.; Ghoniem, N. Thermodynamic Stability Assessment of Oxides, Nitrides, and Carbides in Liquid Sn-25Li, APEX Study, Mech. & Aerospace Engr. Dept. University of California Los Angeles, 2000, UCLA-UCMEP-00-32.

(4) Ren, C. E.; Zhao, M. Q.; Makaryan, T.; Halim, J.; Boota, M.; Kota, S.; Anasori, B.; Barsoum, M. W.; Gogotsi, Yu. Porous Two-Dimensional Transition Metal Carbide (MXene) Flakes for High-Performance Li-Ion Storage. *ChemElectroChem* **2016**, *3*, 689 –693.

(5) Huang, S.; Mochalin, V. N.; Hydrolysis of 2D Transition-Metal Carbides (MXenes) in Colloidal Solutions. *Inorg. Chem.* **2019,** *58*, 1958–1966.

(6) Wei, W.; Ihrfors, C.; Björefors, F.; Nyholm, L. Capacity Limiting Effects for Freestanding, Monolithic TiO_2 Nanotube Electrodes with High Mass Loadings. *ACS Appl. Energy Mater.* **2020**, *3*, 4638–4649.

(7) Borghols, W. J. H.; Lützenkirchen-Hecht, D.; Haake, U.; Chan, W.; Lafont, U.; Kelder, E. M.; van Eck, E. R. H.; Kentgens, A. P. M.; Mulder, F. M.; Wagemaker, M. Lithium Storage in Amorphous TiO_2 Nanoparticles. *J. Electrochem. Soc.* **2010,** *157*: A582-A588.

(8) Li, Z.; Yu, L.; Milligan, C.; Ma, T.; Zhou, L.; Cui, Y.; Qi, Z.; Libretto, N.; Xu, B.; Luo, J. et al. Two-dimensional transition metal carbides as supports for tuning the chemistry of catalytic nanoparticles. *Nat. Commun.* **2018,** *9*: 5258.

(9) Fairely N. CasaXPS Manual 2.3.15 Getting started with CasaXPS. 1.2 rev. ed.; Casa Software Ltd.: England, 2009; pp 1–177.
